# A Novel Fluorescence-Based Microplate Assay for High-Throughput Screening of hSULT1As Inhibitors

**DOI:** 10.3390/bios14060275

**Published:** 2024-05-27

**Authors:** Xiaoting Niu, Yufan Fan, Liwei Zou, Guangbo Ge

**Affiliations:** Shanghai Frontiers Science Center of TCM Chemical Biology, Institute of Interdisciplinary Integrative Medicine Research, Shanghai University of Traditional Chinese Medicine, Shanghai 201203, China; 22021487@shutcm.edu.cn (X.N.); 12022196@shutcm.edu.cn (Y.F.); chemzlw@shutcm.edu.cn (L.Z.)

**Keywords:** human sulfotransferase 1As (hSULT1As), fluorogenic substrate, microplate-based assay, high-throughput screening (HTS), hSULT1As inhibitors

## Abstract

Human sulfotransferase 1As (hSULT1As) play a crucial role in the metabolic clearance and detoxification of a diverse range of endogenous and exogenous substances, as well as in the bioactivation of some procarcinogens and promutagens. Pharmacological inhibiting hSULT1As activities may enhance the in vivo effects of most hSULT1As drug substrates and offer protective strategies against the hSULT1As-mediated bioactivation of procarcinogens. To date, a fluorescence-based high-throughput assay for the efficient screening of hSULT1As inhibitors has not yet been reported. In this work, a fluorogenic substrate (**HN-241**) for hSULT1As was developed through scaffold-seeking and structure-guided molecular optimization. Under physiological conditions, **HN-241** could be readily sulfated by hSULT1As to form **HN-241 sulfate**, which emitted brightly fluorescent signals around 450 nm. **HN-241** was then used for establishing a novel fluorescence-based microplate assay, which strongly facilitated the high-throughput screening of hSULT1As inhibitors. Following the screening of an in-house natural product library, several polyphenolic compounds were identified with anti-hSULT1As activity, while pectolinarigenin and hinokiflavone were identified as potent inhibitors against three hSULT1A isozymes. Collectively, a novel fluorescence-based microplate assay was developed for the high-throughput screening and characterization of hSULT1As inhibitors, which offered an efficient and facile approach for identifying potent hSULT1As inhibitors from compound libraries.

## 1. Introduction

Mammalian sulfotransferases (SULTs), a crucial class of conjugative enzymes, catalyze the transfer of a sulfuryl moiety (−SO_3_H) from the donor 3′-phosphoadenosine-5′-phosphosulfate (PAPS) to various compounds bearing a hydroxyl or amino group [1,2]. Compared to SULTs substrates, sulfated metabolites usually possess increased water solubility that significantly facilitates the excretion of hydrophobic substances via urine or bile. In mammals, SULTs play a crucial role in the metabolic clearance and detoxification defense of endogenous substances (e.g., steroid hormones and catecholamines) and xenobiotics (e.g., clinical drugs, food components, and environmental chemicals) [3]. In humans, a total of 11 functional SULT isoenzymes have been identified. Among all identified human SULTs, the human sulfotransferase 1As (hSULT1As) subfamily has recently drawn increasing attention due to its crucial roles in catalyzing chemically diverse phenolic compounds, including hormones, phenolic phytochemicals, and environmental chemicals, as well as clinical drugs. The hSULT1As subfamily has three members, including hSULT1A1 (phenol sulfotransferase), hSULT1A2 (phenol sulfotransferase), and hSULT1A3 (catecholamine sulfotransferase) [4,5]. hSULT1A1 exhibits a preference for metabolizing small phenolic compounds, estrogen, and 3,3′-diiodothyronine [6,7]. hSULT1A2 also prefers to catalyze the *O*-sulfation of diverse phenols, but it commonly displays relatively weak affinities compared to hSULT1A1 [8,9]. hSULT1A3 is favored for catalyzing the sulfation of catecholamines (such as dopamine, adrenaline, and norepinephrine) [10,11,12]. Notably, three hSULT1A isoforms share a high degree of amino acid sequence homology (>93%), making the substrate spectrum of hSULT1As highly overlapped [11].

Given the critical role of hSULT1As in the metabolic clearance, detoxification, and bioactivation of endogenous substances and exogenous phenolics, modulating hSULT1As activities may strongly affect the in vivo effects of hSULT1As substrates. On the one hand, the pharmacological inhibition of hSULT1As can elevate the circulating exposure levels and oral bioavailability of hSULT1As drug substrates (such as ethinylestradiol and aspirin) and the bioactive phenolics (such as flavonoids and resveratrol) in foods or herbal medicines, thereby improving their therapeutic effect. For instance, ascorbic acid (a competitive inhibitor of hSULT1As) could significantly boost the bioavailability of ethinylestradiol by approximately 50% [13,14,15]. On the other hand, hSULT1As also catalyze several procarcinogens (e.g., benzylic alcohols, aromatic hydroxylamines, and *N*-hydroxyaristolactams) to form reactive metabolites, which may lead to cell damage or organ injury via covalent binding DNA or proteins [16]. Therefore, hSULT1As potent inhibitors can block the hSULT1As-triggered activation of procarcinogens, offering protective strategies against chemically induced cell damage [13,14,17,18,19,20].

Over the past decade, high-performance liquid chromatography (HPLC) and liquid chromatography–tandem mass spectrometry (LC-MS/MS) have been routinely used for the screening of hSULT1As inhibitors by utilizing some known physiological substrates and drug substrates for hSULT1As [21,22,23]. *p*-Nitrophenol (pNP) and dopamine have been widely used for screening hSULT1A1 and hSULT1A3 inhibitors, respectively. However, these methods are labor-intensive, time-consuming, and incapable of the high-throughput screening of hSULT1As inhibitors. Radioactively labeled substrates (such as ^35^S-PAPS) have also been reported for monitoring hSULT1As activity and screening hSULT1As inhibitors [24,25], but such methods require radioactive labeling, thereby raising environmental and safety concerns. In sharp contrast, enzyme-activatable fluorogenic substrates have garnered a growing degree of attention owing to their inherent advantages, such as ultrahigh sensitivity, facile operation, low cost, and practicability for high-throughput screening of the modulators for target enzyme(s) [26,27]. Nevertheless, the practical fluorogenic substrate for high-throughput screening hSULT1As inhibitors has not yet been reported.

In this work, a fluorogenic substrate (termed **HN-241**) for hSULT1As was developed through scaffold-seeking and structure-guided molecular optimizations. Firstly, eight common polycyclic fluorophores bearing one conserved phenolic group were collected to investigate their metabolic potential for hSULT1As, while the spectroscopic variations following *O*-sulfation were also investigated. Fluorophore **B** (4-hydroxy-*N*-butyl-1,8-naphthalimide) stood out as a fluorogenic scaffold for hSULT1As, due to its sulfated metabolite exhibiting distinctly spectroscopic variations and a well-separated emissive band (with the Δλ_em_ of 110 nm). The sulfated metabolite of fluorophore **B** could be quantified at 450 nm and was almost unaffected by the fluorescence signals from the substrate. Next, a suite of 4-hydroxy-1,8-naphthalimide (**4-HN**) derivatives was synthesized to obtain an ideal substrate for hSULT1As, with high conversion rates. Following structural optimizations and phenotyping assays, 4-hydroxy-*N*-ethyl-1,8-naphthalimide (**HN-241**) was finally identified as a good fluorogenic substrate for hSULT1As. A new fluorescence-based microplate assay was then established for the high-throughput screening and characterization of hSULT1As inhibitors, while several polyphenolic compounds were identified as potent hSULT1As inhibitors from an in-house natural compound library.

## 2. Materials and Methods

### 2.1. Reagents and Instruments

4-Bromo-1,8-naphthalic anhydride, 4-fluoroaniline, sulfolane, sulfurtrioxide pyridine complex, tetrakis(triphenylphosphine)palladium, and MgCl_2_ were procured from Sinopharm Chemical Reagent (Shanghai, China). Fluorophores and **4-HN** derivatives were synthesized by our laboratory. Dithiothreitol (DTT) was purchased from Tansoole (Shanghai, China). 3′-Phosphoadenosine-5′-phosphosulfate (PAPS) was acquired from SyncoZymes (Shanghai, China). The recombinant hSULT1A1, hSULT1A2, and hSULT1A3 were expressed in *Escherichia coli* BL21 (DE3). Natural product compounds were primarily obtained from Bidepharm (Shanghai, China), Nature Standard (Shanghai, China), PufeiDe (Chengdu, China), Makclin Biochemical (Shanghai, China), Meilun Bio. (Dalian, China), and Aladdin (Shanghai, China). Quantitative analysis of hSULT1As metabolic profiles of fluorophores and **4-HN** derivatives were detected using a high-performance liquid chromatograph (Nexera LC-40, Shimadzu, Japan), with PDA tandem fluorescence dual detectors with a ShimNex HE C18-EP column (5 μm, 2.1 × 150 mm; Shimadzu, Japan). **HN-241** and its sulfated metabolite (**HN-241 sulfate**) were analyzed by a SCIEX Triple TOF 5600 mass spectrometer (AB SCIEX, Foster City, CA, USA). The BioTek Synergy H1 multi-mode microplate reader (Agilent, Santa Clara, CA, USA) was utilized to determine the emission spectra of fluorogenic substrates and to screen hSULT1As inhibitors.

### 2.2. Expression and Purification of Recombinant Human SULT1As

The expression and purification of hSULT1As followed previously described methods [28,29]. The cDNAs for hSULT1A1, hSULT1A2, and hSULT1A3 linked a 6-histidine tag at the N-terminus with the TEV site, and these were chemically synthesized by GenScript. Using the restriction sites NdeI (5′ end) and HindIII (3′ end), the cDNA constructs were cloned into the expression vector, pET29a (+). Competent *Escherichia coli* (*E. coli*) strain BL21 (DE3) cells were transformed with the pET29a (+)-SULTs vectors. The expression of hSULT1As was induced in the autoinduction method at 18 °C. Following 36 h of induction, *E. coli* was harvested via centrifugation. The *E. coli* precipitate was resuspended in lysis buffer (pH 8.0, 25 mM Tris-HCl, 150 mM NaCl, 1 mM DTT, 1 mM PMSF, 0.1 mg/mL lysozyme, and 25 U/mL Super Nuclease) and lysed for 30 min. The *E. coli* precipitate was then lysed via sonication on ice, and the debris was removed through centrifugation at 18,000 rpm at 4 °C for 30 min. The supernatant was collected and purified using Ni-NTA agarose. The three hSULT1A isoforms were further purified on a Superdex 200 10/300 GL column in protein storage buffer (pH 7.4, 25 mM HEPES, 150 mM NaCl, and 0.5 mM TCEP). Fractions containing hSULT1As were collected and stored at −80 °C.

### 2.3. hSULT1As Reaction Phenotyping Assay

The incubation system consisted of Tris-HCl buffer (pH 7.4, 50 mM), MgCl_2_ (5 mM), DTT (200 μM), recombinant hSULT1As (29 pmol), and each fluorophore or **4-HN** derivative (10 μM) [30]. After pre-incubation at 37 °C for 3 min, the reaction was initiated by adding an excess of PAPS (200 μM). Following 1 h incubation, the reaction was terminated with the addition of an equal volume of ice-cold acetonitrile. After centrifugation at 20,000× *g* for 20 min at 4 °C, the supernatant was collected for HPLC analysis. The mobile phase consisted of 10 mM aqueous ammonium acetate (A) and acetonitrile (B) at a flow rate of 0.4 mL/min. Gradient elution was performed under the following conditions: 0–2.0 min, 5% B; 2.0–10.0 min, 5–95% B; 10.0−12.0 min, 95% B; 12.0–12.5 min, 95–5% B; and 12.5–15.0 min, 5% B.

### 2.4. Spectroscopic Changes in the Tested Fluorophores Catalyzed by hSULT1As

The spectroscopic changes in these fluorophores following *O*-sulfation were investigated. The incubation system was the same as that used in Section 2.3. The emission spectra of eight fluorophores in the presence and absence of three hSULT1A isoforms were determined using BioTek Synergy H1.

### 2.5. Fluorescence Properties of HN-241 and Its Sulfated Metabolite

The fluorescence quantum yield is defined as the ratio of the number of photons emitted to the number of photons absorbed, which is a key parameter in fluorescence spectroscopy that describes the efficiency of the fluorescence process. The fluorescence quantum yields (*Φ*) of **HN-241** and **HN-241 sulfate** were measured using quinine sulfate in 50 mM aqueous H_2_SO_4_ (*Φ* = 0.55) as the standard, and calculated according to the following Equation (1).

(1) $$Y_{u}=\frac{Y_{s}\;\times \;F_{u}\;\times \;A_{s}}{F_{s}\;\times \;A_{u}}$$

“A” represents the absorbance at the excitation wavelength, “F” denotes the integral fluorescence intensity, and the subscripts “s” and “u” refer to the standard and sample, respectively.

### 2.6. Enzymatic Kinetics of hSULT1As-Catalyzed HN-241 4-O-Sulfation

The enzymatic kinetic parameters of hSULT1As-catalyzed **HN-241** 4-*O*-sulfation were measured by incubating increasing concentrations of **HN-241** with each of the hSULT1As for a certain period. The fitting equations included Equation (2), the Michaelis–Menten equation, and Equation (3) for enzyme kinetics with substrate inhibition. Related kinetic parameters were fitted by GraphPad Prism 8.0 (San Diego, CA, USA).

(2) $$V=\frac{V_{\max}\;\times \;[S]}{K_{m}+[S]}$$

(3) $$V=\frac{V_{\max}\;\times \;[S]}{K_{m}+[S](1+\frac{\left[S\right]}{K_{i}})}$$

[S] refers to the concentration of **HN-241**, *V*_max_ is the maximum rate of hSULT1As-catalyzed **HN-241** 4-*O*-sulfation, *K*_m_ is the substrate concentration at the half *V*_max_, and *K*_i_ represents the dissociation constant for the binding of the substrate to the inhibitory site.

### 2.7. Molecular Docking

The 3D structure of **HN-241** was optimized by energy minimization using the ChemBio3D Ultra 14.0 device (Waltham, MA, USA). Crystal structures of hSULT1A1, hSULT1A2, and hSULT1A3 (PDB ID: 1AQU, 2Z5F, and 2A3R) were retrieved from the Protein Data Bank (http://www.rcsb.org, accessed on 15 April 2024). All receptors were pretreated using QuickPrep in MOE (Montreal, QC, Canada) and then docked with **HN-241** in AutoDockTools-1.5.6 (La Jolla, CA, USA). Discovery Studio (version 2020, Dassault Systèmes, San Diego, CA, USA) and PyMOL (version 2.4, Schrödinger, LLC., New York, NY, USA) were utilized to measure the distances between the active site of hSULT1As and the phenolic group of **HN-241**, as depicted in the protein–ligand interactions.

### 2.8. Screening and Characterization of hSULT1As Inhibitors

A novel fluorescence-based microplate assay for screening hSULT1As inhibitors was developed. An in-house natural product library was employed to access the inhibitory potential of three hSULT1A isoforms, using **HN-241** as the probe substrate. The reaction system was composed of Tris-HCl buffer (pH 7.4, 50 mM), MgCl_2_ (5 mM), DTT (200 μM), hSULT1As, **HN-241**, and test compounds at final concentrations of 1 μM or 10 μM. The hSULT1As concentration, **HN-241** concentration, and reaction time were optimized for each isoform. A positive inhibitor (quercetin) was also applied under the same conditions. Following a pre-incubation for 3 min at 37 °C, the reaction was initiated by the addition of an excess of PAPS (200 μM). The BioTek Synergy H1 was used to monitor the reaction in real-time at 37 °C for 15 min, with the excitation wavelength (λ_ex_) and emission wavelength (λ_em_) at 350 nm and 450 nm, respectively. The residue activities were calculated according to Equation (4) and visualized in scatter plots using Origin 2021.

(4) $$\mathrm{Residue}\;\mathrm{activity}\;(\%)=\frac{I_{inhibitor}}{I_{control}}\times 100\%$$

I_inhibitor_ represents the fluorescence intensity of **HN-241 sulfate** in the presence of the inhibitor. I_control_ refers to the fluorescence intensity of **HN-241 sulfate**, without inhibitor (DMSO only).

### 2.9. Determination of Half-Maximal Inhibitory Concentration (IC_50_)

The reaction system is described in Section 2.7. The IC_50_ values were determined by incubating hSULT1As and **HN-241** with increasing concentrations of the newly identified inhibitors in the incubation system. The control group was performed using DMSO only, in the absence of any inhibitor. The residual activities of all tested compounds were measured in real time using BioTek Synergy H1 within 15 min. The IC_50_ values were calculated by nonlinear regression using GraphPad Prism 8.0 software (GraphPad Software, Inc., La Jolla, CA, USA).

### 2.10. Statistical Analysis

Unless otherwise noted, all kinetic analyses and inhibitor screenings were performed in triplicate. All data were expressed as the mean ± SD. The scatter plots for screening inhibitors were analyzed utilizing Origin 2021 (Northampton, MA, USA). *K*_m_ and IC_50_ values were obtained with GraphPad Prism 8.0 software.

## 3. Results and Discussion

### 3.1. Seeking an Optimum Scaffold for Constructing hSULT1As Fluorogenic Substrates

**Figure 1:**
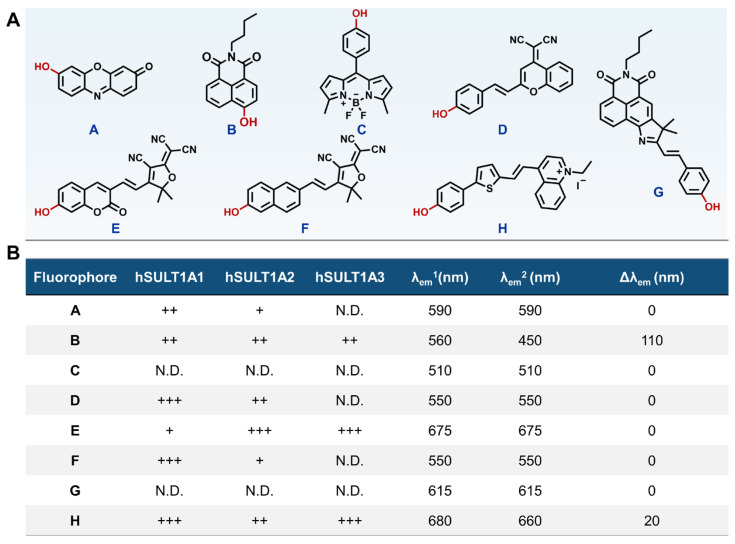
(**A**) Chemical structures of eight candidate fluorophores. (**B**) Sulfation rates and spectroscopic variations of eight fluorophores catalyzed by hSULT1As. λ_em_^1^ denotes the emission wavelength of each tested fluorophore, without hSULT1As. λ_em_^2^ denotes the emission wavelength of eight fluorophores in the presence of hSULT1As. Δλ_em_ denotes the spectroscopic changes of fluorophores in the presence and absence of hSULT1As. “+” shows the peak area of the sulfated metabolites of each fluorophore detected by HPLC, ranging from 0 to 10,000. “++” and “+++” denote ranges from 10,000 to 50,000, and greater than 50,000, respectively. “N.D.” means not detected.

Firstly, eight common fluorophores bearing one phenolic group were collected on the basis of the substrate preferences of hSULT1As. These fluorophores were then incubated with each hSULT1A isoenzyme to seek an optimum fluorogenic scaffold (Schemes S1–S6 and Figures S1–S20) [31,32,33,34,35,36,37,38,39,40]. The results demonstrated that fluorophores **C** and **G** could not be sulfated by any tested hSULT1A isozymes, while the other tested fluorophores could be sulfated by two or three hSULT1As. Although fluorophores **A**, **D**, **E**, and **F** could be sulfated by hSULT1As, their fluorescence signals were dramatically decreased, and none of the new emission peaks were observed following *O*-sulfation (Figure 1 and Figure S21), suggesting that the *O*-sulfates of these fluorophores displayed weak fluorescence emission. **H** exhibited the highest *O*-sulfation rate in hSULT1As, but the spectroscopic analysis revealed that its *O*-sulfated metabolite showed a maximal emission wavelength of 660 nm, exhibiting a tiny blue shift (only ~20 nm) compared to that of the substrate (680 nm). Such tiny changes in emission wavelength posed a great challenge for differentiating the substrate and its *O*-sulfated metabolite by fluorescence detection without chromatographic isolation. In sharp contrast, fluorophore **B** could be readily sulfated by hSULT1As and generated a single fluorogenic metabolite, which showed a remarkable fluorescence enhancement (~100-fold) around 450 nm following sulfation. These finding revealed that fluorophore **B** is a promising fluorogenic scaffold for constructing hSULT1As fluorogenic substrates, but its *O*-sulfation rates should be further improved.

### 3.2. Structural Optimizations of 4-HN to Improve Sulfation Rate

**Figure 2:**
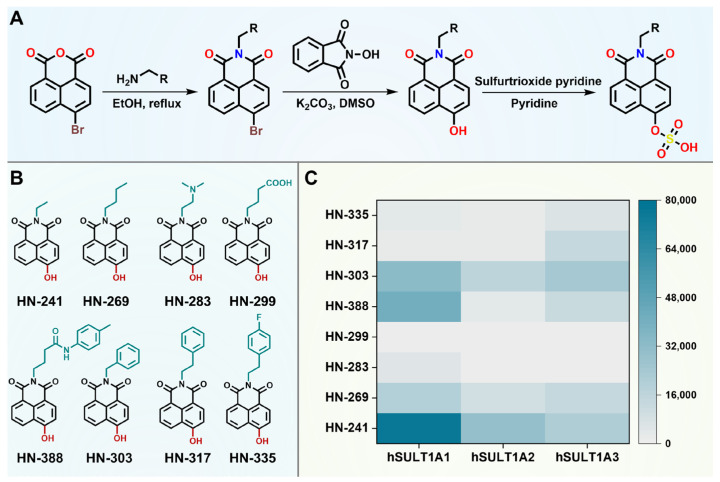
(**A**) Synthetic route of **4-HN** derivatives and their sulfates. (**B**) Chemical structures of **4-HN** derivatives. (**C**) The fluorescence intensity of the sulfated metabolites of the **4-HN** derivatives catalyzed by hSULT1As.

Next, to obtain an ideal substrate for hSULT1As, with high reactivity and desirable affinity, a suite of 4-hydroxyl-1,8-naphthalimide (**4-HN**) derivatives was designed and synthesized via introducing various aliphatic or aromatic substitutions at the *N*-site (Figure 2A,B and Figure S22–S35) [41,42]. As shown in Figure 2C, the sulfation rates of these derivatives varied significantly with the hydrophobicity and the molecular size of the north substituents. Compared to **HN-269**, **HN-283** and **HN-299** (bearing a hydrophilic group in the north part) demonstrated very slow *O*-sulfation rates in hSULT1As. Meanwhile, **HN-317** and **HN-335** (bearing a bulky substituent in the north part) also exhibited very slow *O*-sulfation rates in hSULT1As. **HN-388** (bearing a bulky substituent) could be rapidly metabolized in hSULT1A1, but this agent was scarcely metabolized in hSULT1A2. Conversely, **HN-241** (bearing an *N*-ethyl substituent) displayed the highest *O*-sulfation rate, making it a favorable substrate for sensing enzyme activity and the high-throughput screening of hSULT1As inhibitors. These findings confirmed that **4-HN** derivatives bearing a small hydrophobic group are good substrates for hSULT1As.

### 3.3. Fluorescence Properties of HN-241 and Its Sulfated Metabolite

**Figure 3:**
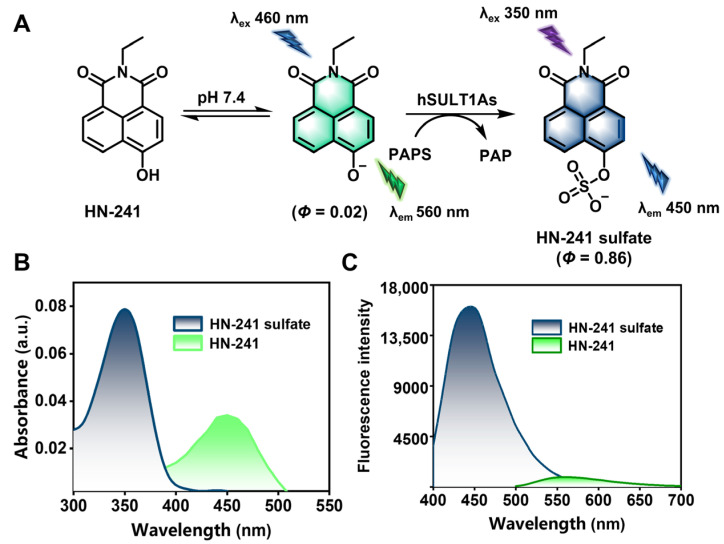
(**A**) Chemical structures of **HN-241** and its sulfated metabolite (**HN-241 sulfate**). (**B**,**C**) Absorbance and emission spectra of **HN-241** (10 μM) and **HN-241 sulfate** (10 μM).

**HN-241 sulfate** was then synthesized and fully characterized by NMR and high-resolution mass spectrometry (Scheme S7, Figures S36–S39) [43]. Subsequently, the fluorescence properties of both **HN-241** and **HN-241 sulfate** were then investigated. Under physiological conditions, **HN-241** displayed a maximal absorption peak at 450 nm, while the absorption peak of **HN-241 sulfate** was blue-shifted to 350 nm (Figure 3). **HN-241** (*Φ* = 0.02) emitted a weak fluorescent signal around 560 nm, whereas **HN-241 sulfate** (*Φ* = 0.86) produced a stable and brightly fluorescent signal around 450 nm, with a high signal-to-noise ratio (SNR). These findings point out that **HN-241** acts as a favorable fluorogenic substrate for hSULT1As.

### 3.4. Enzymatic Kinetics of hSULT1As-Catalyzed HN-241 4-O-Sulfation

**Figure 4:**
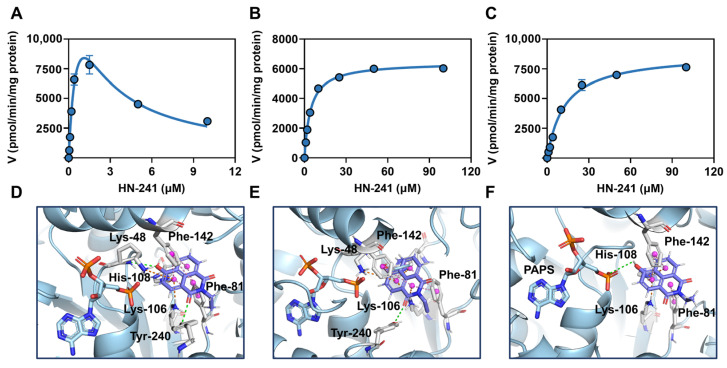
Kinetic plots of **HN-241**-catalyzed 4-*O*-sulfation by hSULT1A1 (**A**), hSULT1A2 (**B**), and hSULT1A3 (**C**). The interactions of **HN-241** in hSULT1A1 (**D**), hSULT1A2 (**E**), and hSULT1A3 (**F**). Green line: conventional hydrogen bond; magenta line: π–π stacked; yellow line: π–cation.

As shown in Figure 4A–C and Figure S40, and Table 1, hSULT1A1-catalyzed **HN-241** 4-*O*-sulfation displayed substrate inhibition kinetics, while **HN-241** 4-*O*-sulfation in both hSULT1A2 and hSULT1A3 followed the classical Michaelis–Menten kinetics. For hSULT1A1, **HN-241** demonstrated a high binding affinity (*K*_m_ = 0.91 µM) and intrinsic clearance rate (*Cl*_int_ = 24,265.93 µL/min/mg protein). For hSULT1A2 and hSULT1A3, favorable binding affinity (*K*_m_ values of 4.47 µM and 12.90 µM, for hSULT1A2 and hSULT1A3, respectively) and high **HN-241** 4-*O*-sulfation rates were observed, with the *V*_max_ values of 6441.00 pmol/min/mg protein and 8814.00 pmol/min/mg protein, for hSULT1A2 and hSULT1A3, respectively. The combination of favorable affinity and fluorescence properties of **HN-241** *O*-sulfation provides an essential foundation for the high-throughput screening of hSULT1As inhibitors.

### 3.5. Binding Modes of HN-241 in hSULT1As

Subsequently, docking simulations were conducted to further explore the binding modes of **HN-241** in hSULT1As. **HN-241** could be well-docked into the catalytic cavity of each tested hSULT1A isoenzyme, while the hSULT1A1-substrate complex displayed the lowest binding energy (Figure S41, and Table S1). In all tested hSULT1A isoenzymes, the naphthalene ring of **HN-241** could interact with Phe-81 and Phe-142 via π–π stacked interactions (Figure 4D–F). The oxygen atom in the naphthalene ring of **HN-241** created a hydrogen bond with Tyr-240 in hSULT1A1 and Tyr-240 in hSULT1A2, while such interactions were not observed in hSULT1A3. More importantly, it was observed that the phenolic hydroxyl of **HN-241** formed an additional hydrogen bond with Lys-48 of hSULT1A1, which could partially explain the high binding affinity of hSULT1A1 in **HN-241** when compared with other two hSULT1As.

### 3.6. High-Throughput Screening and Characterization of hSULT1As Inhibitors

**Figure 5:**
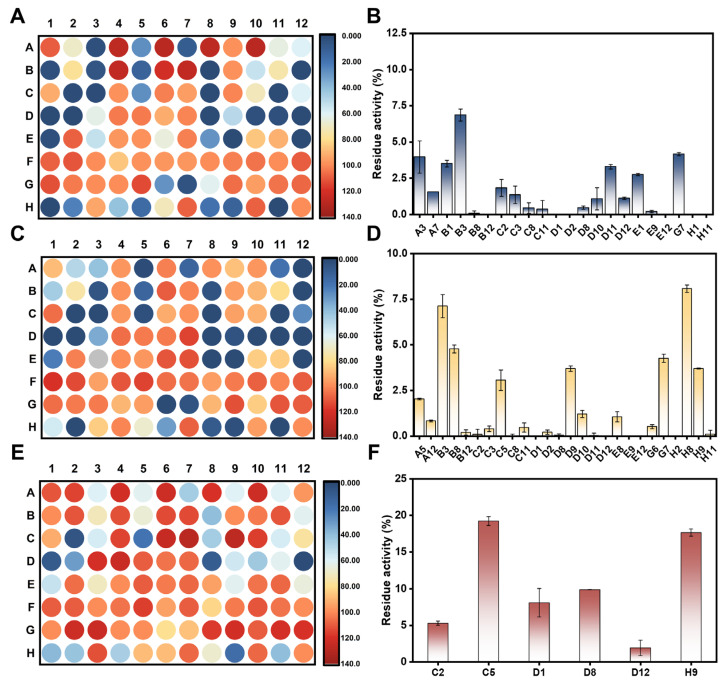
Scatter plots of the residue activities of 94 natural compounds inhibiting hSULT1A1 (**A**), hSULT1A2 (**C**), and hSULT1A3-catalyzed (**E**) **HN-241** 4-*O*-sulfation. A gradient from red to blue indicates residual activity from high to low. The identified potent hSULT1A1 (**B**) and hSULT1A2 (**D**) inhibitors, with a residue activity less than 10%, and the strong hSULT1A3 (**F**) inhibitors, with residue activity less than 20%.

A novel fluorescence-based high-throughput screening method was developed utilizing **HN-241** as the substrate. Prior to inhibitor screening, the conditions for the high-throughput screening of hSULT1A inhibitors were further optimized, including substrate concentration, hSULT1As concentration, and reaction time (Figure S42 and Table S2). A known inhibitor of hSULT1As (quercetin) was also used to validate the high-throughput screening method (Figure S43), showing results consistent with those of previous reports [44]. Next, we screened the anti-hSULT1As potentials of 94 compounds from an in-house natural product library. Of all tested compounds, 22 compounds exhibited potent anti-hSULT1A1 activity, showing the residual activity of less than 10% at the dose of 1 μM (Figure 5A,B, and Table S3). A total of 26 compounds exhibited effective anti-hSULT1A2 activity, showing the residual activity of less than 10% at the dose of 10 μM (Figure 5C,D). In contrast, only four compounds showed substantial anti-hSULT1A3 activity at the dose of 10 μM (Figure 5E,F). It was observed that most of the identified potent hSULT1A1 inhibitors also showed strong to moderated anti-hSULT1A2 effects, indicating that these two isoenzymes shared the overlapped inhibitor spectra. By contrast, most newly identified hSULT1A1 inhibitors showed weak antti-hSULT1A3 effects, which agreed well with the previous studies [17,45]. As shown in Figure S44, the newly identified inhibitors of hSULT1As could significantly prevent the formation of **HN-241 sulfate** (determined by LC-FD), suggesting that these newly identified hSULT1As inhibitors are real inhibitors rather than competitive binders.

**Figure 6:**
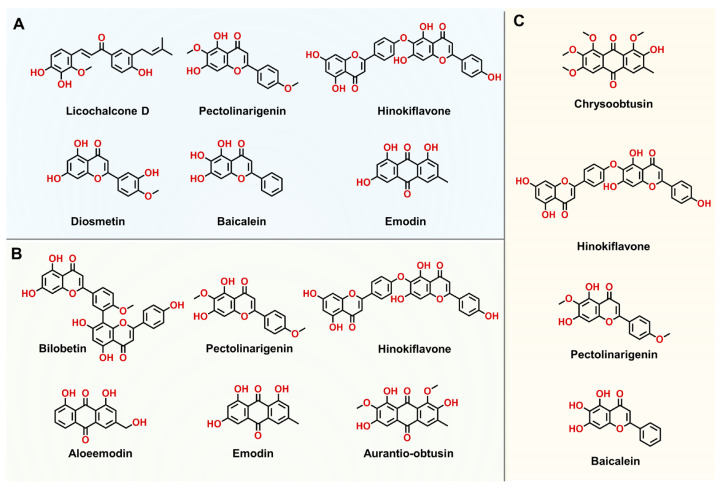
Structures of the newly identified inhibitors of hSULT1A1 (**A**), hSULT1A2 (**B**), and hSULT1A3 (**C**).

**Figure 7:**
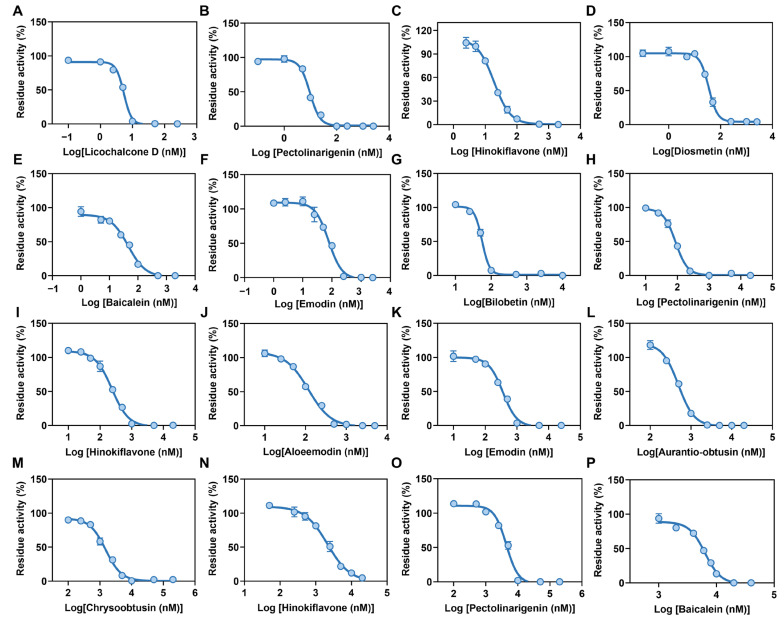
(**A**–**F**) Dose-inhibition curves of licochalcone D, pectolinarigenin, hinokiflavone, diosmetin, baicalein, and emodin against hSULT1A1-catalyzed **HN-241** 4-*O*-sulfation. (**G**–**L**) Dose-inhibition curves of bilobetin, pectolinarigenin, hinokiflavone, aloeemodin, emodin, and aurantio-obtusin against hSULT1A2. (**M**–**P**) The dose-inhibition curves of chrysoobtusin, hinokiflavone, pectolinarigenin, and baicalein against hSULT1A3.

We further plotted the dose-inhibition curves for these newly identified hSULT1As inhibitors. Pectolinarigenin and hinokiflavone emerged as the potent inhibitors of all three hSULT1A isoforms. Licochalcone D and pectolinarigenin emerged as the most potent inhibitors against hSULT1A1, with the IC_50_ values of 5.37 ± 0.18 nM and 9.52 ± 0.39 nM, respectively. Hinokiflavone, diosmetin, baicalein, and emodin also acted as potent inhibitors against hSULT1A1, with IC_50_ values ranging from 18.88 nM to 76.53 nM (Figure 6A and Figure 7A–F, and Table 2). Bilobetin and pectolinarigenin exhibited strong anti-hSULT1A2 effects, with IC_50_ values of 55.47 ± 1.30 nM and 87.24 ± 3.16 nM, respectively (Figure 6B and Figure 7G–L). Chrysoobtusin, hinokiflavone, pectolinarigenin, and baicalein showed relatively strong anti-hSULT1A3 activity, with IC_50_ values of ranging from 1515 nM to 6319 nM (Figure 6C and Figure 7M–P). In addition, we also performed inhibition kinetic assays for the potent inhibitor bilobetin against hSULT1A2-catalyzed **HN-241** 4-*O*-sulfation. Bilobetin could potently inhibit hSULT1A2-catalyzed **HN-241** 4-*O*-sulfation, in a mixed inhibition manner (Figure S45).

## 4. Conclusions

In summary, a novel fluorescence-based biochemical assay was established for the high-throughput screening and characterizing of hSULT1As inhibitors. Firstly, a desirable fluorogenic substrate (**HN-241**) for hSULT1As was developed through scaffold-seeking and structure-guided molecular optimization, which could be readily sulfated by hSULT1As to generate a single brightly fluorogenic metabolite. **HN-241** was then used for developing a novel fluorescence-based microplate assay for the high-throughput screening and characterizing of hSULT1As inhibitors. Compared to HPLC or LC-MS/MS-based assays, the fluorescence-based microplate assay offered a superior combination of high sensitivity, facile operation, and real-time monitoring. Following the screening of an in-house natural product library, pectolinarigenin and hinokiflavone were identified as potent inhibitors against three hSULT1A isoenzymes. Collectively, this work devises a favorable fluorogenic substrate (**HN-241**) for hSULT1As, which was then used to developed a practical tool for the high-throughput screening and characterization of hSULT1As inhibitors.

## Figures and Tables

**Table 1:** Kinetic parameters of the **HN-241**-catalyzed 4-*O*-sulfation in hSULT1As.

**Enzyme**	***V*** **_max_** **(pmol/min/mg Protein)**	***K*** **_m_** **(μM)**	***Cl*** **_int_** **(** **μL/min/mg Protein** **)**	***K*** **_i_** **(μM)**
hSULT1A1	22,082.00 ± 4426.00	0.91 ± 0.24	24,265.93	1.37 ± 0.40
hSULT1A2	6441.00 ± 72.43	4.47 ± 0.20	1440.94	--
hSULT1A3	8814.00 ± 242.80	12.90 ± 1.16	683.26	--

**Table 2:** The IC_50_ values of the newly identified SUL1As inhibitors.

**Enzyme**	**Compound**	**MW**	**IC_50_ (** **n** **M)**
hSULT1A1	licochalcone D	354.40	5.37 ± 0.18
	pectolinarigenin	314.29	9.52 ± 0.39
	hinokiflavone	538.46	18.88 ± 1.17
	diosmetin	300.27	34.73 ± 1.34
	baicalein	270.24	45.19 ± 3.91
	emodin	270.24	76.53 ± 5.28
hSULT1A2	bilobetin	552.49	55.47 ± 1.30
	pectolinarigenin	314.29	87.24 ± 3.16
	hinokiflavone	538.46	110.50 ± 14.70
	aloeemodin	270.24	117.70 ± 7.20
	emodin	270.24	359.80 ± 22.57
	aurantio-obtusin	330.29	491.90 ± 15.18
hSULT1A3	chrysoobtusin	358.35	1515.00 ± 79.14
	hinokiflavone	538.46	2127.00 ± 182.10
	pectolinarigenin	314.29	4364.00 ±252.20
	baicalein	270.24	6319.00 ± 194.90

## Data Availability

The dataset is available on request from the authors.

## Supplementary Materials

The following supporting information can be downloaded at: https://www.mdpi.com/article/10.3390/bios14060275/s1. **Scheme S1**. Synthetic route of 4-HN derivatives. Reagents and conditions: (a) amine compounds, ethanol, reflux, 8 h; (b) 2-hydroxyisoindoline-1,3-dione, K2CO3, DMSO, 80 °C, 6 h; **Scheme S2**. Synthetic route of candidate fluorophore D; **Scheme S3**. Synthetic route of candidate fluorophore E. Reagents and conditions: (a) malononitrile, CH3COONH4; (b) Raney nickel, formic acid (88%), 80 °C, 5 h; (c) malononitrile, sodium ethoxide, EtOH, reflux, 6 h; (d) EtOH, piperidine, reflux, overnight; **Scheme S4**. Synthetic route of candidate fluorophore F; **Scheme S5**. Synthetic route of fluorophore G. Reagents and conditions: (a) n-butylamine, ethanol, reflux, 8 h; (b) hydrazine hydrate, 2-methoxy ethanol, reflux, 4 h; (c) 3-methyl-2-butanone, H2SO4, reflux, 4 h; (d) 4-hydroxybenzaldehyde, acetonitrile, piperidine, acetic acid, reflux; **Scheme S6**. Synthetic route of fluorophore H. Reagents and conditions: (a) 4-hydroxyphenylboronic acid, tetrakis(triphenylphosphine)palladium, K2CO3 (2M), 1,4-dioxane, reflux, 10 h; (b) 1-ethyl-4-methylquinolin-1-ium, acetonitrile, piperidine, acetic acid, reflux; **Scheme S7**. Synthetic route of HN-241 sulfate; **Figure S1**. 1H NMR spectrum of fluorophore B; **Figure S2**. 13C NMR spectrum of fluorophore B; **Figure S3**. 1H NMR spectrum of fluorophore D; **Figure S4**. 13C NMR spectrum of fluorophore D; **Figure S5**. 1H NMR spectrum of 1-3; **Figure S6**. 13C NMR spectrum of 1-3; **Figure S7**. 1H NMR spectrum of 1-4; **Figure S8**. 13C NMR spectrum of 1-4; **Figure S9**. 1H NMR spectrum of fluorophore E; **Figure S10**. 13C NMR spectrum of fluorophore E; **Figure S11**. 1H NMR spectrum of fluorophore F; **Figure S12**. 13C NMR spectrum of fluorophore F; **Figure S13**. 1H NMR spectrum of 1-7; **Figure S14**. 13C NMR spectrum of 1-7; **Figure S15**. 1H NMR spectrum of fluorophore G; **Figure S16**. 13C NMR spectrum of fluorophore G; **Figure S17**. 1H NMR spectrum of 1-8; **Figure S18**. 13C NMR spectrum of 1-8; **Figure S19**. 1H NMR spectrum of fluorophore H; **Figure S20**. 13C NMR spectrum of fluorophore H; **Figure S21**. The emission spectra of fluorophore A, B, D, E, F, and H catalyzed by hSULT1As; **Figure S22**. 1H NMR spectrum of HN-241; **Figure S23**. 13C NMR spectrum of HN-241; **Figure S24**. 1H NMR spectrum of HN-283; **Figure S25**. 13C NMR spectrum of HN-283; **Figure S26**. 1H NMR spectrum of HN-299; **Figure S27**. 13C NMR spectrum of HN-299; **Figure S28**. 1H NMR spectrum of HN-388; **Figure S29**. 13C NMR spectrum of HN-388; **Figure S30**. 1H NMR spectrum of HN-303; **Figure S31**. 13C NMR spectrum of HN-303; **Figure S32**. 1H NMR spectrum of HN-317; **Figure S33**. 13C NMR spectrum of HN-317; **Figure S34**. 1H NMR spectrum of HN-335; **Figure S35**. 13C NMR spectrum of HN-335; **Figure S36**. 1H NMR spectrum of HN-241 sulfate; **Figure S37**. 13C NMR spectrum of HN-241 sulfate; **Figure S38**. The HPLC profiles of HN-241 and its sulfation metabolite (HN-241 sulfate); **Figure S39**. The MS/MS spectra of HN-241 and its sulfation metabolite (HN-241 sulfate); **Figure S40**. The standard curve of HN-241 sulfate using LC-FD; **Figure S41**. The molecular docking of HN-241 (deep bule) in hSULT1A1, hSULT1A2, and hSULT1A3; **Figure S42**. The liner relationship of fluorescence intensity at 450 nm for different reaction times (0–30 min) for hSULT1A1 (A), hSULT1A2 (B), and hSULT1A3 (C) catalyzed HN-241 4-O-sulfation; **Figure S43**. Dose-inhibition curves of quercetin against hSULT1A1 (A), hSULT1A2 (B), hSULT1A3-catalyzed (C) HN-241 4-O-sulfation; **Figure S44**. (A) The chromatograms of HN-241 in the presence of PAPS and hSULT1A1 with or without the newly identified hSULT1A1 inhibitor. (B) The chromatograms of HN-241 in the presence of PAPS and hSULT1A2 with or without the newly identified hSULT1A2 inhibitor. (C) The chromatograms of HN-241 in the presence of PAPS and hSULT1A3 with or without the newly identified hSULT1A3 inhibitor. HN-241 sulfate detected by LC-FD with excitation wavelength (λex) and emission wavelength (λem) at 350 nm and 450 nm; **Figure S45**. (A) Inhibition kinetics of bilobetin against hSULT1A2. (B) The second plot of slope from the Lineweaver-Burk plot for inhibition of bilobetin against hSULT1A2; **Table S1**. The molecular docking of HN-241 in hSULT1As; **Table S2**. The reaction conditions of screening system for hSULT1As inhibitors; **Table S3**. Inhibitory activity of 94 natural compounds against hSULT1As-catalyzed HN-241 4-O-sulfation.
