# Supplementary material for: A Novel Fluorescence-Based Microplate Assay for High-Throughput Screening of hSULT1As Inhibitors
Source: Biosensors (Basel). 2024 May 27;14(6):275. doi: 10.3390/bios14060275 (PMC11202169; doi:10.3390/bios14060275)
Supplement: Supplementary file 1 [file biosensors-14-00275-s001.zip › biosensors-2986377-supplementary.pdf]

**Supporting Information**

*for*

# A Novel Fluorescence-Based Microplate Assay for High-Throughput Screening of hSULT1As Inhibitors

XiaoTing Niu+ [1], YuFan Fan+ [1], LiWei Zou [1], and GuangBo Ge [1,*]

[1] Shanghai Frontiers Science Center of TCM Chemical Biology, Institute of Interdisciplinary Integrative Medicine Research, Shanghai University of Traditional Chinese Medicine, Shanghai 201203, China.; 22021487@shutcm.edu.cn (X.-T. N.); 12022196@shutcm.edu.cn (Y.-F. F.); chemzlw@shutcm.edu.cn (L.-W. Z.); geguangbo@shutcm.edu.cn (G.-B. G.)

[*] Correspondence: geguangbo@shutcm.edu.cn (G.-B. G.)

**Synthesis of fluorophores**

**General synthetic procedure of 4-HN derivatives**

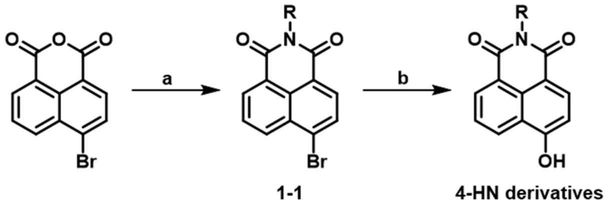


**Scheme S1.** Synthetic route of **4-HN** derivatives. Reagents and conditions: (a) amine compounds, ethanol, reflux, 8 h; (b) 2-hydroxyisoindoline-1,3-dione, $K_2CO_3$, DMSO, 80°C, 6 h.

**4-HN** derivatives were synthesized according to the previously reported references [34, 42−43]. 4-bromo-1,8-naphthalic anhydride (5.0 mmol) and each amine compound (6.0 mmol) were dissolved in 20 mL EtOH and refluxed for 8 h. After cooling, the precipitate was filtered and washed with EtOH to afford the intermediate product **1-1** without further purification. Next, the crude intermediate and $K_2CO_3$ (6.0 mmol) were mixed in DMSO (20 mL) and stirred at 80°C for 6 h after adding 2-hydroxyisoindoline-1,3-dione (6.0 mmol). Subsequently, the reaction solution was poured into the ice water. After adjusting the pH to 5~6, the generated precipitate was filtered and washed with water. Finally, the crude compound was further purified by a silica gel column chromatograph ($CH_2Cl_2$/$CH_3OH$).

Fluorophore **B**: Yellow solid (yield = 80.2%). $^1$H NMR (600 MHz, DMSO-$d_6$) $\delta$ 11.89 (s, 1H), 8.53 (dd, $J$ = 8.4, 1.2 Hz, 1H), 8.47 (dd, $J$ = 7.3, 1.2 Hz, 1H), 8.36 (d, $J$ = 8.2 Hz, 1H), 7.76 (t, $J$ = 7.8 Hz, 1H), 7.17 (d, $J$ = 8.1 Hz, 1H), 4.06 – 3.99 (m, 2H), 1.60 (p, $J$ = 7.5 Hz, 2H), 1.34 (h, $J$ = 7.4 Hz, 2H), 0.92 (t, $J$ = 7.4 Hz, 3H). $^{13}$C NMR (151 MHz, DMSO-$d_6$) $\delta$ 164.15, 163.49, 160.73, 134.03, 131.61, 129.66, 129.37, 126.10, 122.86, 122.30, 113.09, 110.45, 30.22, 20.29, 14.20.

**Synthesis of fluorophore D**

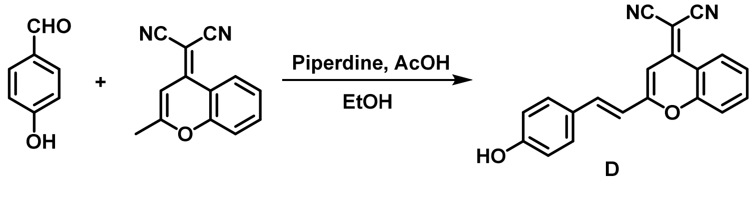

**Scheme S2.** Synthetic route of candidate fluorophore **D**.

4-Hydroxybenzaldehyde (128 mg, 1.05 mmol), 2-(2-methyl-4*H*-chromen-4-ylidene) malononitrile (208 mg, 1 mmol), piperidine (0.1 mL), and acetic acid (0.1 mL) were heated and refluxed in acetonitrile for 12 h. After the reaction was complete, the mixture was concentrated under reduced pressure conditions. The crude product was purified by a silica gel column (DCM/MeOH = 50: 1, v/v) and further recrystallized with DCM/PE to acquire brick red powder (151.3 mg, 48.5%). $^{1}$H NMR (400 MHz, DMSO-$d_6$) $\delta$ 8.72 (d, $J$ = 8.3 Hz, 1H), 7.91 (t, $J$ = 7.8 Hz, 1H), 7.78 (d, $J$ = 8.4 Hz, 1H), 7.72 – 7.55 (m, 4H), 7.26 (d, $J$ = 15.9 Hz, 1H), 6.94 (s, 1H), 6.85 (d, $J$ = 8.4 Hz, 2H). $^{13}$C NMR (101 MHz, DMSO-$d_6$) $\delta$ 160.51, 159.37, 153.35, 152.49, 139.76, 135.75, 130.84, 126.57, 126.53, 125.06, 119.49, 117.88, 117.58, 116.53, 116.51, 116.36, 106.18, 59.53.

**Synthesis of fluorophore E**

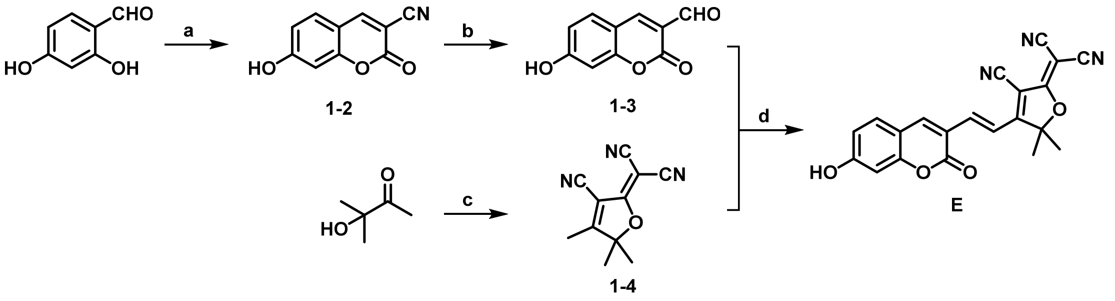


**Scheme S3.** Synthetic route of candidate fluorophore **E**. Reagents and conditions: (a) malononitrile, $CH_3COONH_4$; (b) Raney nickel, formic acid (88%), 80°C, 5 h; (c) malononitrile, sodium ethoxide, EtOH, reflux, 6 h; (d) EtOH, piperidine, reflux, overnight.

Synthesis of **1-2**. The synthetic method of coumarin probe referred to a previously reported scheme [44]. In brief, a mixture of 2,4-dihydroxybenzaldehyde (500 mg, 3.62 mmol) and malononitrile (358 mg, 5.42 mmol) in $CH_3COONH_4$ aqueous solution was stirred for 5 hours. Then, diluted hydrochloric acid (2 M) was added to adjust the pH to 4~5 for another 1 h at 75°C. After the reaction, the solid was filtered out to obtain compound 1-2 without further purification for the next reactions.

Synthesis of **1-3**. Compound **1-2** reacted with excess Raney nickel in formic acid at 80°C for 5 h, accompanied by the change of suspended solids from orange to bright

yellow. The reaction solution was added to the excess ice water and filtered. Compound **1-3** was obtained after the filter cake was extracted by ethyl acetate under reflux conditions. $^{1}$H NMR (600 MHz, DMSO-*d*$_6$) $\delta$ 9.97 (s, 1H), 8.58 (d, *J* = 1.8 Hz, 1H), 7.82 (dd, *J* = 8.7, 1.4 Hz, 1H), 6.89 – 6.86 (m, 1H), 6.78 (t, *J* = 2.0 Hz, 1H). $^{13}$C NMR (151 MHz, DMSO-*d*$_6$) $\delta$ 188.26, 165.46, 160.23, 158.06, 147.70, 133.85, 117.51, 115.04, 111.34, 102.76.

Synthesis of **1-4**. Under ice bath conditions, 3-hydroxy-3-methyl-2-butanone (5.1 g, 50 mmol), malononitrile (7.9 g, 120 mmol), and sodium ethoxide (510 mg, 7.5 mmol) were dissolved in anhydrous ethanol and refluxed for 6 h. After the reaction was complete, the mixture was cooled overnight in a 4°C refrigerator. The filter cake was obtained by filtered directly, while the filtrate was extracted through EA. After merging all the crude products, the white crystal product (5.8 g, 58.3%) was acquired and purified through a silica gel column (DCM/PE = 5: 1). $^{1}$H NMR (600 MHz, Chloroform-*d*) $\delta$ 2.39 (s, 3H), 1.65 (s, 6H). $^{13}$C NMR (101 MHz, Chloroform-*d*) $\delta$ 182.40, 175.13, 110.99, 110.34, 108.94, 104.86, 99.69, 58.62, 24.40, 14.18.

Synthesis of fluorophore **E**. Compound **1-3** (190 mg, 1 mmol) and **1-4** (219 mg, 1.1 mmol) were dissolved in a mixture solvent (EtOH/piperidine) and refluxed for 12 hours. After the reaction was complete, the red powder (214 mg, yield = 57.7%) was obtained through filtrating and recrystallizing with MeOH. $^{1}$H NMR (600 MHz, DMSO-*d*$_6$) $\delta$ = 8.66 (s, 1H), 7.74 (s, 2H), 7.66 (d, *J* = 8.6 Hz, 1H), 6.93-6.88 (m, 1H), 6.81-6.78 (m, 1H), 1.81 (s, 6H). $^{13}$C NMR (151 MHz, DMSO-*d*$_6$) $\delta$ = 177.39, 175.77, 165.04, 159.24, 156.59, 149.64, 143.42, 132.29, 117.11, 116.95, 115.23, 113.21, 112.41, 112.39, 111.26, 102.67, 100.26, 99.47, 54.66, 25.88.

**Synthesis of fluorophore F**

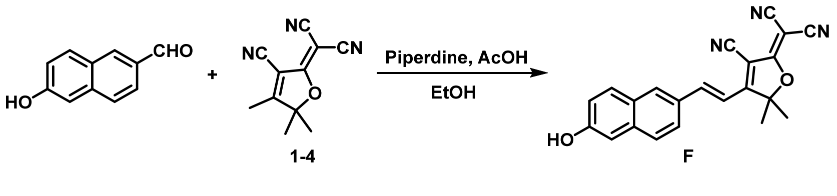


**Scheme S4.** Synthetic route of candidate fluorophore **F**.

6-Hydroxy-2-naphthaldehyde (172 mg, 1 mmol), **1-4** (219 mg, 1.1 mmol), piperidine (0.1 mL), and acetic acid (0.1 mL) were dissolved in EtOH and refluxed for 12 hours. After the reaction was complete, the red powder (280 mg, yield = 79.2%) was obtained through filtrating and recrystallizing with EA/PE. $^1$H NMR (400 MHz, DMSO-$d_6$) $\delta$ 10.34 (s, 1H), 8.32 (d, $J$ = 1.8 Hz, 1H), 8.10 (d, $J$ = 16.3 Hz, 1H), 7.97 (dd, $J$ = 8.9, 1.8 Hz, 1H), 7.91 (d, $J$ = 8.8 Hz, 1H), 7.80 (d, $J$ = 8.8 Hz, 1H), 7.26 (d, $J$ = 16.3 Hz, 1H), 7.21 – 7.15 (m, 2H), 1.82 (s, 6H). $^{13}$C NMR (101 MHz, DMSO-$d_6$) $\delta$ 177.73, 175.89, 158.70, 148.70, 137.33, 133.63, 131.75, 129.53, 127.79, 127.73, 124.53, 120.19, 114.26, 113.33, 112.49, 111.67, 109.83, 99.75, 98.18, 54.25, 25.64.

**Synthesis of fluorophore G**

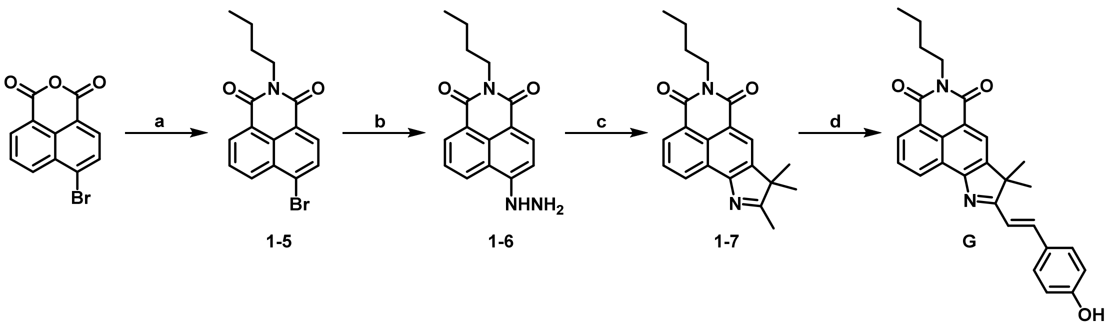


**Scheme S5.** Synthetic route of fluorophore **G**. Reagents and conditions: (a) *n*-butylamine, ethanol, reflux, 8 h; (b) hydrazine hydrate, 2-methoxy ethanol, reflux, 4 h; (c) 3-methyl-2-butanone, $H_2SO_4$, reflux, 4 h; (d) 4-hydroxybenzaldehyde, acetonitrile, piperidine, acetic acid, reflux.

Synthesis of **1-5**. A mixture of 4-bromo-1,8-naphthalic anhydride (5.54 g, 20.0 mmol) and butylamine (2.5 mL, 25.0 mmol) in ethanol (200 mL) was stirred and refluxed for 8 h. After cooling to room temperature, the solids are precipitated, filtered, and washed with ice EtOH to obtain light yellow solids (4.7 g, 71.1%) without purification.

Synthesis of **1-6**. Compound **1-5** (3.32 g, 10.0 mmol) was dissolved in 2-methoxyethanol (40 mL) and heated (120°C) until dissolved. When the solution became clear, hydrazine hydrate (3.5 ml) was slowly added dropwise to the above mixture. After the dropwise addition, the reaction was refluxed for 4 h. The mixture was then cooled to room temperature, accompanied by the precipitation of a large amount of orange

precipitate. The precipitate was further filtered and washed with ice ethanol to obtain compound **1-6** (2.7 g, 95.4%) without further purification.

Synthesis of **1-7**. Compound **1-6** (1.44 g, 5.0 mol) was dissolved in 3-methyl-2-butanone (20 mL) and then slowly added concentrated $H_2SO_4$ (2.5 mL). The reaction mixture was refluxed under nitrogen protection for 4 h. After the reaction was completed, the crude product was obtained by direct filtration. The crude product was beaten with an appropriate amount of methanol and filtered to obtain a light-yellow powder (1.2 g, 70.5%). $^1$H NMR (600 MHz, Chloroform-*d*) $\delta$ 8.98 (d, $J$ = 8.2 Hz, 1H), 8.68 (dd, $J$ = 7.3, 1.2 Hz, 1H), 8.64 (s, 1H), 7.88 (dd, $J$ = 8.3, 7.3 Hz, 1H), 4.26 – 4.21 (m, 2H), 2.66 (s, 3H), 1.78 – 1.73 (m, 2H), 1.56 – 1.44 (m, 8H), 1.01 (t, $J$ = 7.4 Hz, 3H). $^{13}$C NMR (151 MHz, DMSO-$d_6$) $\delta$ 195.77, 164.04, 154.62, 143.93, 131.11, 130.01, 128.56, 127.64, 125.47, 124.50, 122.79, 119.19, 55.89, 30.16, 22.12, 20.25, 16.37, 14.20.

Synthesis of fluorophore **G**. Compounds **1-7** (668 mg, 2.0 mmol) and 4-hydroxybenzaldehyde (244 mg, 2.0 mmol) were dissolved and stirred into the absolute acetonitrile (6 ml), followed by piperidine (0.1 mL) and acetic acid (0.1 mL). The mixture was refluxed under nitrogen, until the reaction was completed. The solvent was removed under reduced pressure, and the crude product was purified by a silica gel column (dichloromethane/methanol) to afford the orange product (695.5 mg, 79.4%). $^1$H NMR (600 MHz, DMSO-$d_6$) $\delta$ 8.90 (d, $J$ = 8.2 Hz, 1H), 8.65 (s, 1H), 8.54 (d, $J$ = 7.2 Hz, 1H), 7.98 – 7.91 (m, 2H), 7.75 (d, $J$ = 8.4 Hz, 2H), 7.28 (d, $J$ = 16.1 Hz, 1H), 6.87 (d, $J$ = 8.2 Hz, 2H), 4.08 (t, $J$ = 7.4 Hz, 2H), 1.64 (p, $J$ = 7.5 Hz, 2H), 1.57 (s, 6H), 1.37 (h, $J$ = 7.5 Hz, 2H), 0.94 (t, $J$ = 7.4 Hz, 3H). $^{13}$C NMR (101 MHz, DMSO-$d_6$) $\delta$ 189.86, 164.07, 164.00, 160.21, 155.74, 144.46, 141.33, 131.19, 130.83, 130.20, 128.77, 127.43, 127.17, 125.25, 124.65, 122.78, 118.63, 116.40, 54.48, 30.20, 22.80, 20.27, 14.23.

**Synthesis of fluorophore H**

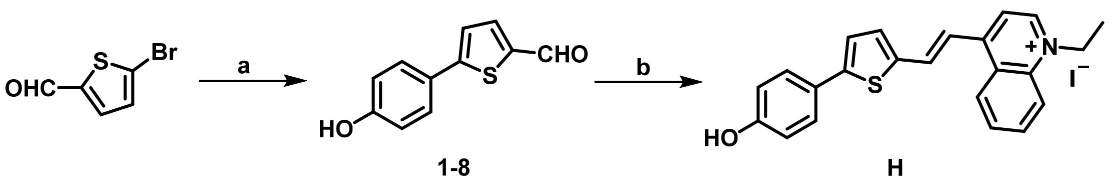


**Scheme S6.** Synthetic route of fluorophore **H**. Reagents and conditions: (a) 4-hydroxyphenylboronic acid, tetrakis(triphenylphosphine)palladium, $K_2CO_3$ (2M), 1,4-dioxane, reflux, 10 h; (b) 1-ethyl-4-methylquinolin-1-ium, acetonitrile, piperidine, acetic acid, reflux.

Synthesis of **1-8**. 5-Bromothiophene-2-carbaldehyde (1.91 g, 10.0 mmol), 4-hydroxyphenylboronic acid (1.38 g, 10.0 mmol), tetrakis(triphenylphosphine)palladium (231 mg, 0.2 mmol), and $K_2CO_3$ (2M, 10.0 mL) were added in 1,4-dioxane (40.0 mL) and refluxed for 10 h. After the reaction was complete, the mixture was extracted by ethyl acetate and concentrated. In the state of heating and stirring, the concentrated mixture was continuously added with petroleum ether. The products were precipitated and filtered after cooling to obtain the brown-yellow crystals (1.2 g, 58.8%). $^1$H NMR (600 MHz, DMSO-$d_6$) $\delta$ 9.99 (s, 1H), 9.86 (s, 1H), 7.98 (d, $J$ = 3.9 Hz, 1H), 7.66 – 7.63 (m, 2H), 7.56 (d, $J$ = 4.0 Hz, 1H), 6.88 – 6.85 (m, 2H). $^{13}$C NMR (151 MHz, DMSO-$d_6$) $\delta$ 184.11, 159.48, 154.19, 140.92, 140.02, 128.32, 123.98, 123.85, 116.58.

Synthesis of fluorophore **H**. Compounds **1-8** (204 mg, 1.0 mmol) and 1-ethyl-4-methylquinolin-1-ium (299 mg, 1.0 mmol) were dissolved and stirred into the absolute acetonitrile (5 ml), followed by piperidine (0.1 mL) and acetic acid (0.1 mL). The mixture was refluxed under nitrogen, until the reaction was completed. The crude product was purified by a silica gel column ($CH_2Cl_2/CH_3OH$=10/1, v/v) to afford the black product (359.4 mg, 74.1%). $^1$H NMR (600 MHz, DMSO-$d_6$) $\delta$ 9.93 (s, 1H), 9.32 (d, $J$ = 6.6 Hz, 1H), 8.96 (d, $J$ = 8.6 Hz, 1H), 8.52 (d, $J$ = 8.9 Hz, 1H), 8.49 – 8.33 (m, 2H), 8.25 (ddd, $J$ = 8.8, 7.1, 1.5 Hz, 1H), 8.10 – 7.98 (m, 1H), 7.91 (dd, $J$ = 15.6, 2.4 Hz, 1H), 7.74 (d, $J$ = 3.9 Hz, 1H), 7.61 (dt, $J$ = 8.8, 2.4 Hz, 2H), 7.52 (t, $J$ = 2.4 Hz, 1H), 6.98 – 6.78 (m, 2H), 4.98 (q, $J$ = 7.2 Hz, 2H), 1.59 (t, $J$ = 7.2 Hz, 3H). $^{13}$C NMR (151 MHz, DMSO-$d_6$) $\delta$ 158.94, 152.65, 149.75, 147.17, 138.98, 138.13, 136.60,

135.53, 134.78, 129.50, 127.77, 127.06, 126.76, 124.55, 124.23, 119.46, 117.63, 116.60, 116.13, 52.42, 15.60.

**Synthetic procedure of 4-HN derivatives**

A series of **4-HN** derivatives was synthesized according to the previous reference reported by our group [34, 42−43].

**HN-241** (yield 75.8%). $^{1}$H NMR (600 MHz, DMSO-$d_6$) $\delta$ 11.86 (s, 1H), 8.53 (dd, $J$ = 8.3, 1.6 Hz, 1H), 8.47 (dt, $J$ = 7.3, 1.6 Hz, 1H), 8.36 (dd, $J$ = 8.3, 1.4 Hz, 1H), 7.79 – 7.73 (m, 1H), 7.16 (d, $J$ = 8.2 Hz, 1H), 4.06 (q, $J$ = 6.9 Hz, 2H), 1.20 (t, $J$ = 7.1 Hz, 3H). $^{13}$C NMR (151 MHz, DMSO-$d_6$) $\delta$ 163.95, 163.28, 160.72, 133.99, 131.56, 129.65, 129.36, 126.08, 122.86, 122.34, 113.13, 110.43, 34.93, 13.70.

**HN-283** (yield 71.8%)**.** $^{1}$H NMR (600 MHz, DMSO-$d_6$) $\delta$ 12.00 (s, 1H), 8.60 (dd, $J$ = 8.3, 1.2 Hz, 1H), 8.52 (dd, $J$ = 7.3, 1.2 Hz, 1H), 8.41 (d, $J$ = 8.2 Hz, 1H), 7.82 (dd, $J$ = 8.3, 7.3 Hz, 1H), 7.20 (d, $J$ = 8.2 Hz, 1H), 4.37 (t, $J$ = 5.8 Hz, 2H), 3.46 (t, $J$ = 5.8 Hz, 2H), 2.92 (s, 6H). $^{13}$C NMR (151 MHz, DMSO-$d_6$) $\delta$ 164.85, 164.06, 161.04, 134.32, 131.82, 129.92, 129.71, 126.23, 122.88, 122.40, 113.09, 110.52, 55.68, 43.33, 35.46.

**HN-299** (yield 76.5%). $^{1}$H NMR (600 MHz, DMSO-$d_6$) $\delta$ 12.02 (s, 1H), 11.85 (s, 1H), 8.53 (dd, $J$ = 8.3, 1.2 Hz, 1H), 8.46 (dd, $J$ = 7.2, 1.2 Hz, 1H), 8.35 (d, $J$ = 8.2 Hz, 1H), 7.76 (dd, $J$ = 8.3, 7.2 Hz, 1H), 7.15 (d, $J$ = 8.2 Hz, 1H), 4.06 (t, $J$ = 7.0 Hz, 2H), 2.29 (t, $J$ = 7.4 Hz, 2H), 1.87 (p, $J$ = 7.2 Hz, 2H). $^{13}$C NMR (151 MHz, DMSO-$d_6$) $\delta$ 174.43, 164.29, 163.61, 160.68, 134.01, 131.59, 129.72, 129.32, 126.07, 122.85, 122.34, 113.14, 110.40, 31.82, 23.59.

**HN-388** (yield 74.3%) $^{1}$H NMR (600 MHz, DMSO-$d_6$) $\delta$ 11.91 (s, 1H), 9.77 (s, 1H), 8.56 – 8.51 (m, 1H), 8.47 (dd, $J$ = 7.3, 1.5 Hz, 1H), 8.36 (dq, $J$ = 8.2, 1.5 Hz, 1H), 7.76 (t, $J$ = 7.8 Hz, 1H), 7.38 (d, $J$ = 8.2 Hz, 2H), 7.15 (d, $J$ = 8.0 Hz, 1H), 7.03 (d, $J$ = 8.1 Hz, 2H), 4.10 (t, $J$ = 7.1 Hz, 2H), 2.35 (t, $J$ = 7.6 Hz, 2H), 2.22 (s, 3H), 1.96 (p, $J$ = 7.4 Hz, 2H). $^{13}$C NMR (151 MHz, DMSO-$d_6$) $\delta$ 170.81, 164.31, 163.63, 160.72, 137.18,

134.06, 132.24, 131.66, 129.73, 129.39, 129.36, 126.10, 122.86, 122.33, 119.51, 113.14, 110.40, 34.52, 24.24, 20.87.

**HN-303** (yield 65.6%). $^{1}$H NMR (400 MHz, DMSO-*d*6) δ 11.94 (s, 1H), 8.55 – 8.51 (m, 1H), 8.47 (dd, *J* = 7.3, 0.9 Hz, 1H), 8.37 (d, *J* = 8.2 Hz, 1H), 7.80 – 7.71 (m, 1H), 7.30 (dt, *J* = 14.8, 7.4 Hz, 4H), 7.22 (d, *J* = 2.3 Hz, 1H), 7.16 (d, *J* = 8.2 Hz, 1H), 5.22 (s, 2H). $^{13}$C NMR (101 MHz, DMSO-*d*6) *δ* 164.22, 163.52, 160.98, 138.14, 134.34, 131.88, 129.75, 129.62, 128.80, 127.97, 127.43, 126.15, 122.89, 122.13, 112.87, 110.53, 43.10.

**HN-317** (yield 69.5%). $^{1}$H NMR (600 MHz, DMSO-*d*6) *δ* 11.88 (s, 1H), 8.53 (dd, *J* = 8.3, 1.2 Hz, 1H), 8.47 (dd, *J* = 7.2, 1.2 Hz, 1H), 8.36 (d, *J* = 8.1 Hz, 1H), 7.76 (dd, *J* = 8.3, 7.2 Hz, 1H), 7.32 – 7.26 (m, 4H), 7.23 – 7.20 (m, 1H), 7.16 (d, *J* = 8.2 Hz, 1H), 4.25 – 4.20 (m, 2H), 2.93 – 2.89 (m, 2H). $^{13}$C NMR (151 MHz, DMSO-*d*6) *δ* 164.03, 163.34, 160.78, 139.33, 134.06, 131.62, 129.66, 129.42, 129.08, 128.93, 126.79, 126.10, 122.86, 122.24, 113.03, 110.46, 41.25, 34.10.

**HN-335** (yield 72.6%). $^{1}$H NMR (600 MHz, DMSO-*d*6) *δ* 11.88 (s, 1H), 8.53 (dd, *J* = 8.3, 1.3 Hz, 1H), 8.46 (dd, *J* = 7.2, 1.2 Hz, 1H), 8.35 (d, *J* = 8.2 Hz, 1H), 7.76 (dd, *J* = 8.3, 7.2 Hz, 1H), 7.33 – 7.24 (m, 2H), 7.15 (d, *J* = 8.2 Hz, 1H), 7.14 – 7.08 (m, 2H), 4.28 – 4.15 (m, 2H), 2.95 – 2.85 (m, 2H). $^{13}$C NMR (151 MHz, DMSO-d6) *δ* 164.04, 163.34, 162.17, 160.91, 160.56, 135.48, 134.08, 131.62, 130.92, 130.87, 129.68, 129.46, 126.07, 122.90, 122.20, 115.67, 115.53, 112.90, 110.49, 41.20, 33.21.

**Synthetic procedure of the sulfation metabolite of HN-241**

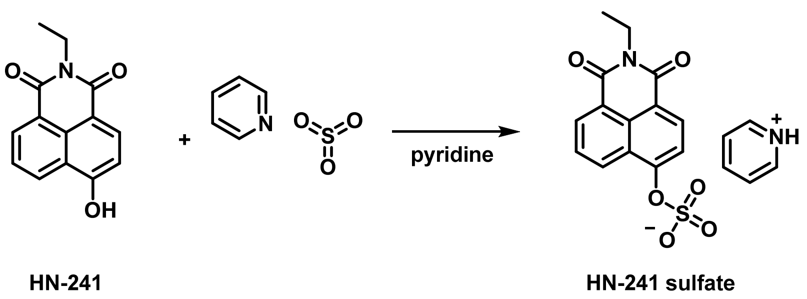


**Scheme S7.** Synthetic route of **HN-241 sulfate**.

**HN-241 sulfate** was synthesized according to the previously reported references [45]. **HN-241** (80 mg, 0.33 mmol) and sulfurtrioxide pyridine complex (127 mg, 0.99 mmol) were dissolved in pyridine and stirred at room temperature for 2 hours. When the reaction was complete, the solvent was removed under reduced pressure. Dichloromethane was added, and the pH was adjusted to 7-8 with sodium bicarbonate to remove insoluble impurities. The organic phase was dried over anhydrous sodium sulfate and then removed under reduced pressure. The crude product was recrystallized from methyl tert-butyl ether to yield a yellow-white solid (38.5 mg, 36.3%). $^{1}$H NMR (600 MHz, DMSO-$d_6$) $\delta$ 8.91 – 8.88 (m, 2H), 8.54 – 8.47 (m, 4H), 8.00 (t, $J$ = 6.3 Hz, 2H), 7.88 – 7.83 (m, 2H), 4.09 (q, $J$ = 7.1 Hz, 2H), 1.21 (t, $J$ = 7.1 Hz, 3H). $^{13}$C NMR (151 MHz, DMSO-$d_6$) $\delta$ 163.90, 163.30, 155.61, 145.28, 143.83, 134.03, 132.82, 131.61, 131.33, 129.52, 129.40, 129.10, 127.20, 126.96, 126.14, 125.02, 122.46, 116.61, 115.76, 110.46, 35.09, 34.94, 13.71, 13.67.

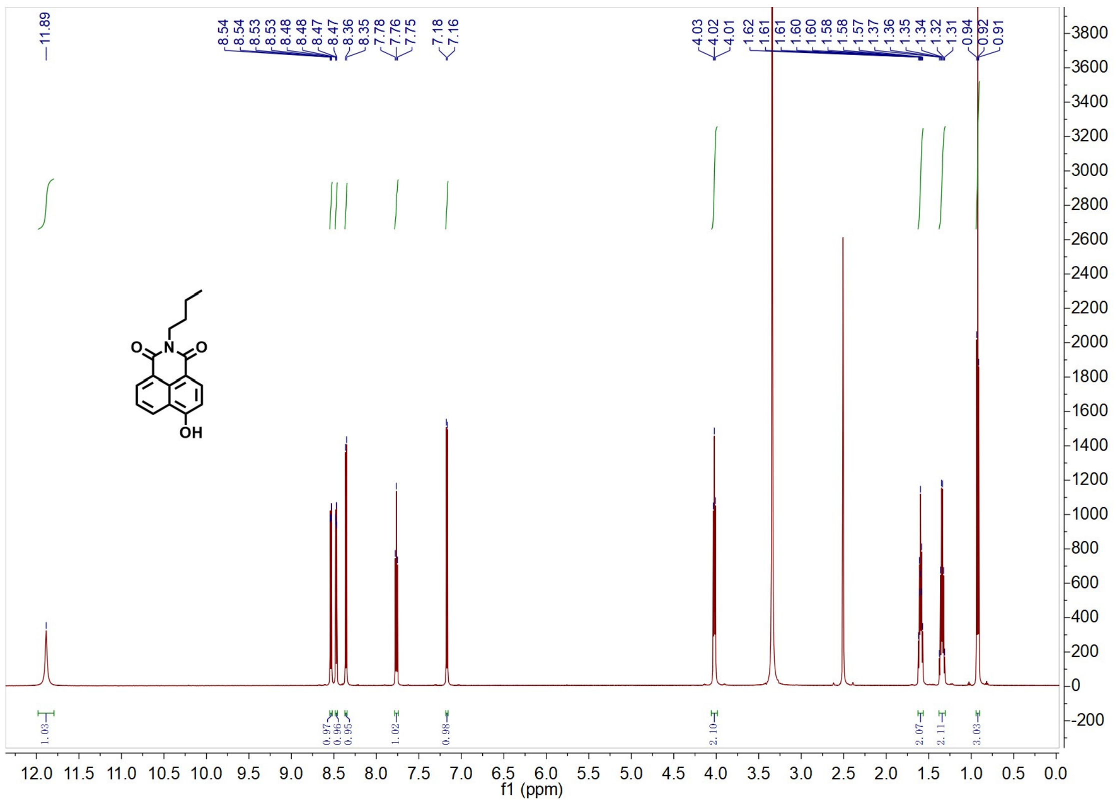

**Figure S1.** $^{1}$H NMR spectrum of fluorophore **B**.

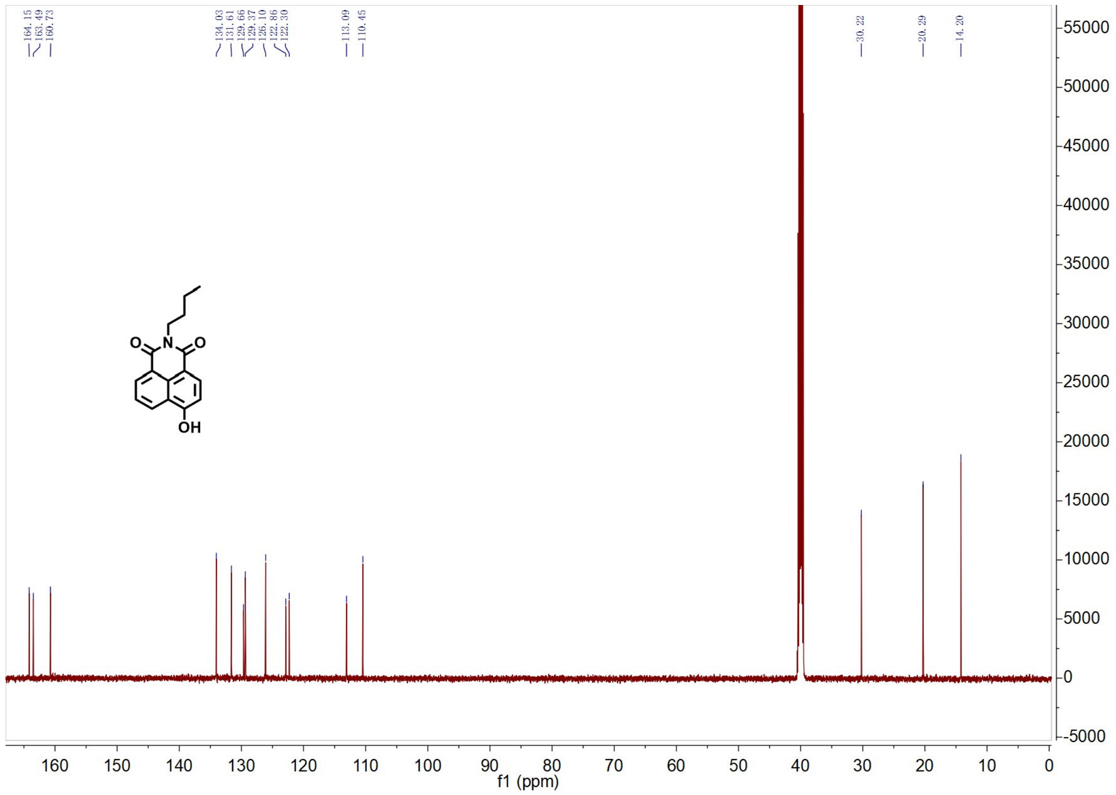

**Figure S2.** $^{13}$C NMR spectrum of fluorophore **B**.

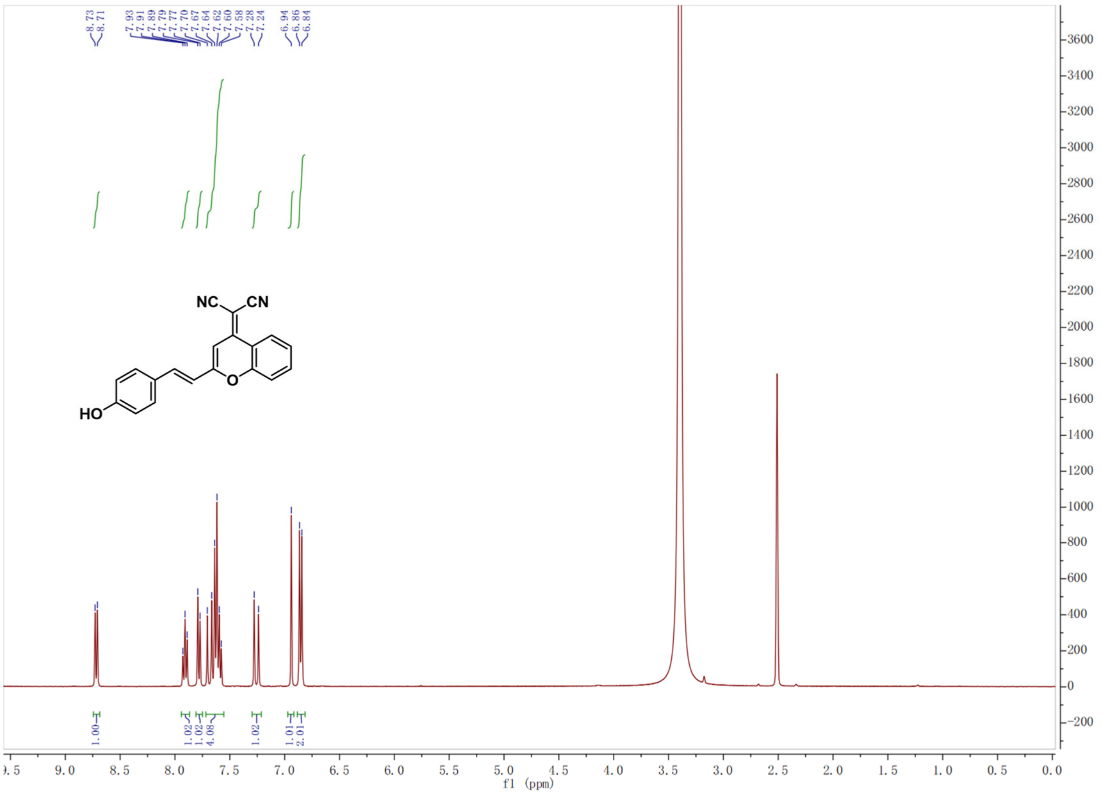

**Figure S3.** $^{1}H$ NMR spectrum of fluorophore **D**.

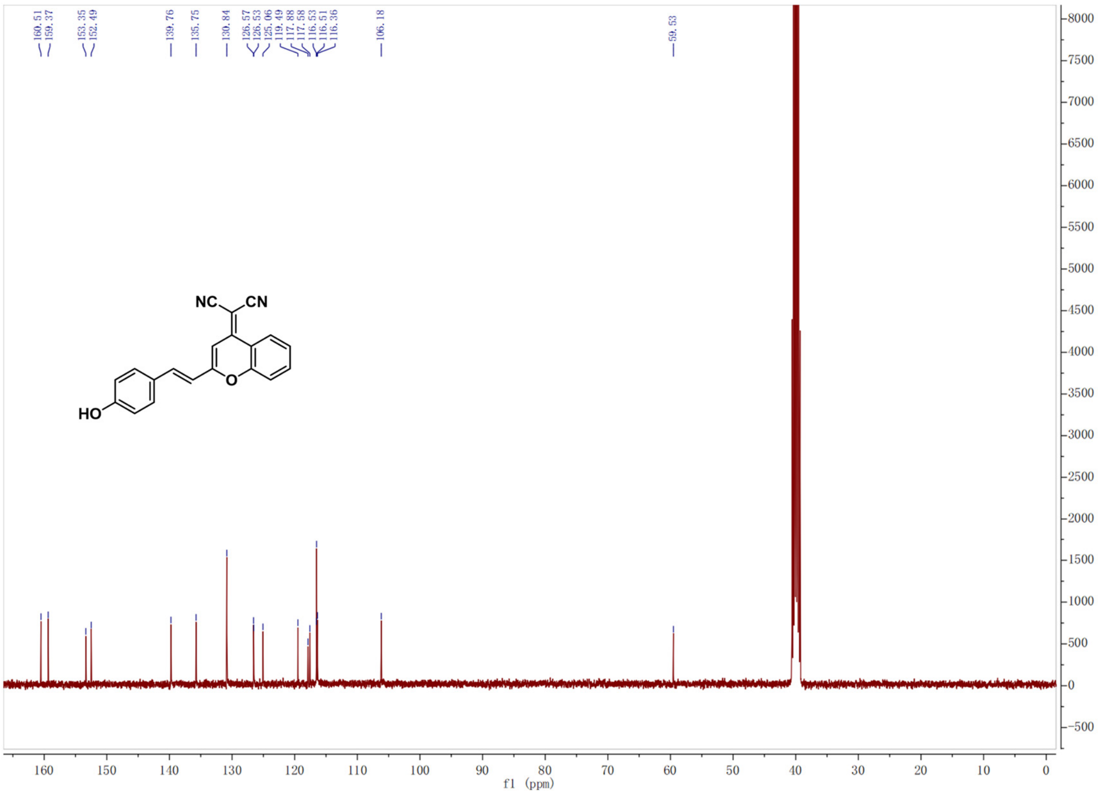

**Figure S4.** $^{13}C$ NMR spectrum of fluorophore **D**.

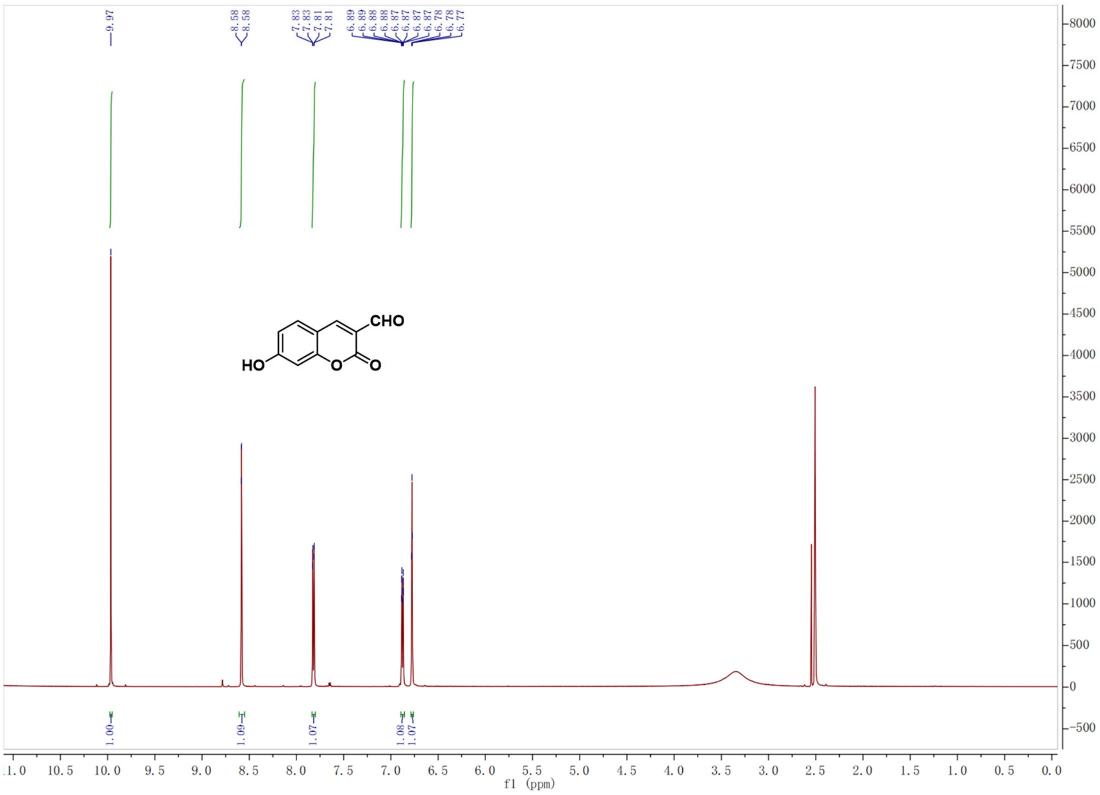


**Figure S5.** $^{1}H$ NMR spectrum of **1-3**.

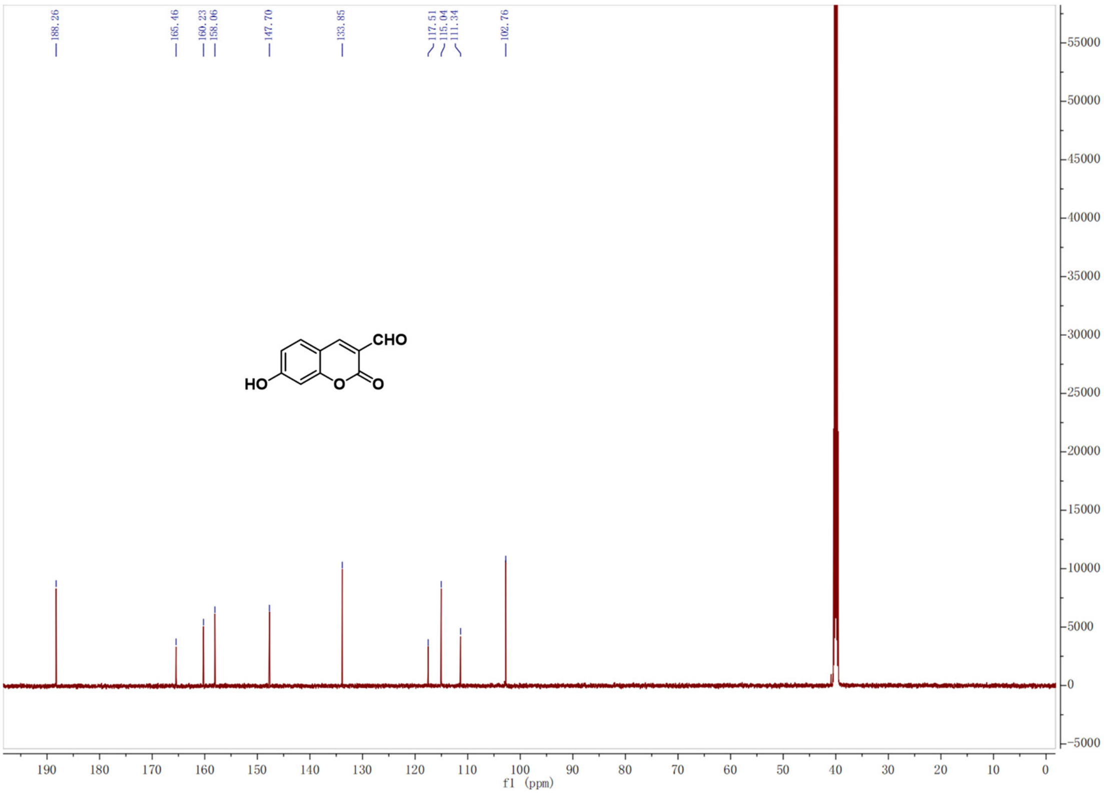


**Figure S6.** $^{13}C$ NMR spectrum of **1-3**.

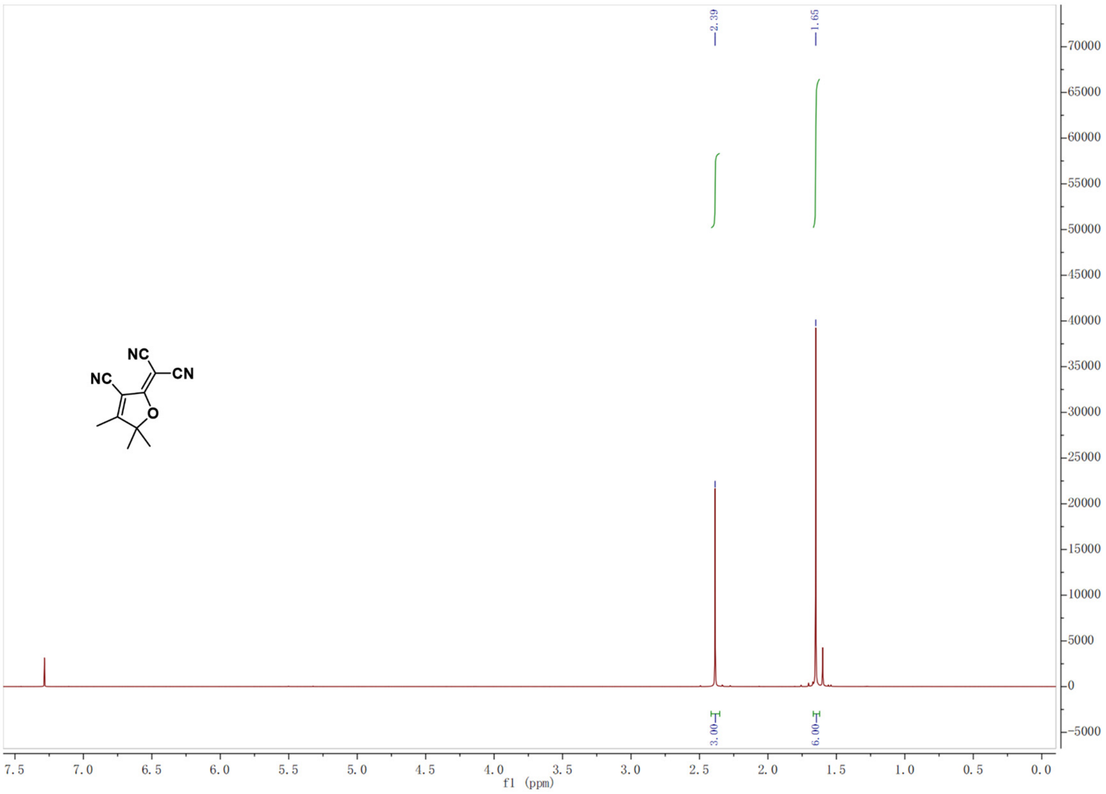


**Figure S7.** $^{1}$H NMR spectrum of **1-4**.

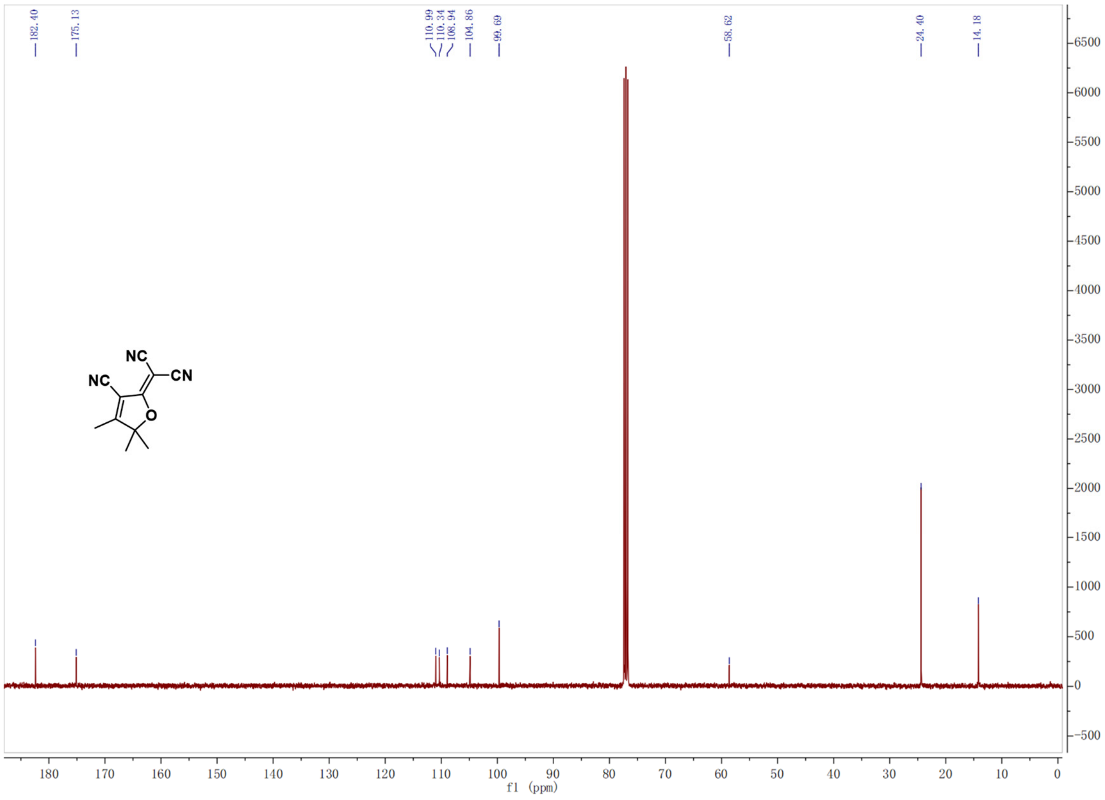


**Figure S8.** $^{13}$C NMR spectrum of **1-4**.

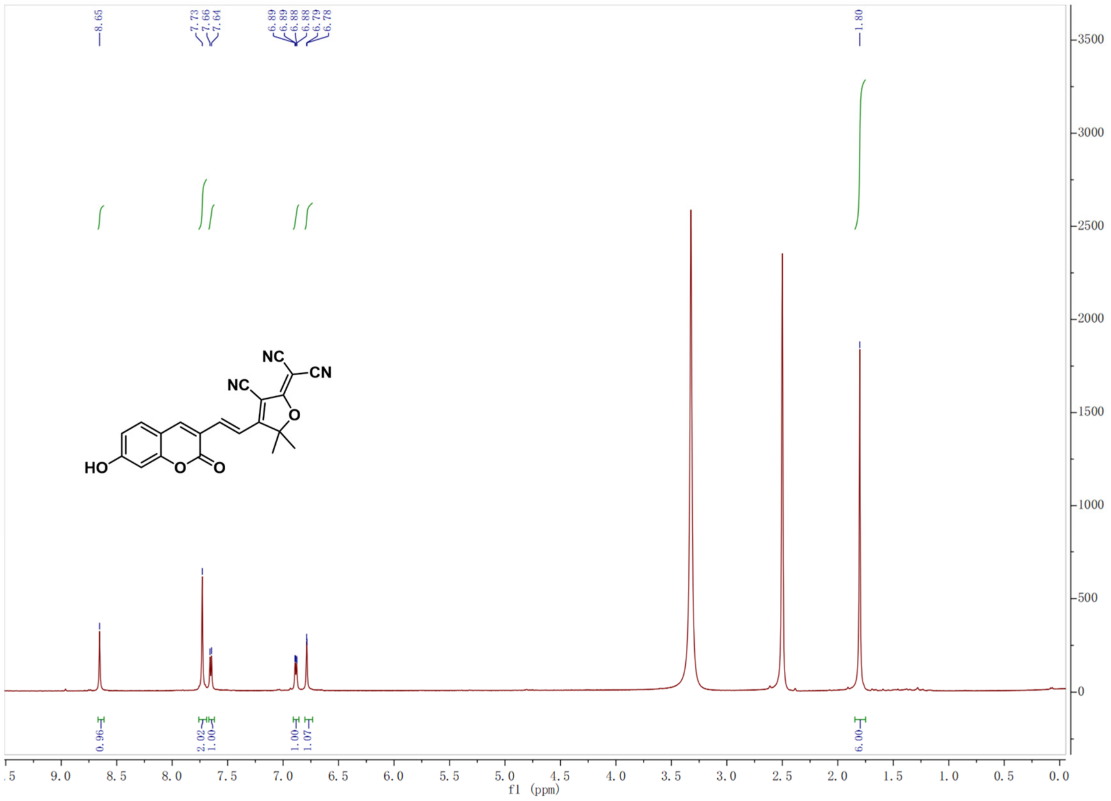

**Figure S9.** $^{1}H$ NMR spectrum of fluorophore **E**.

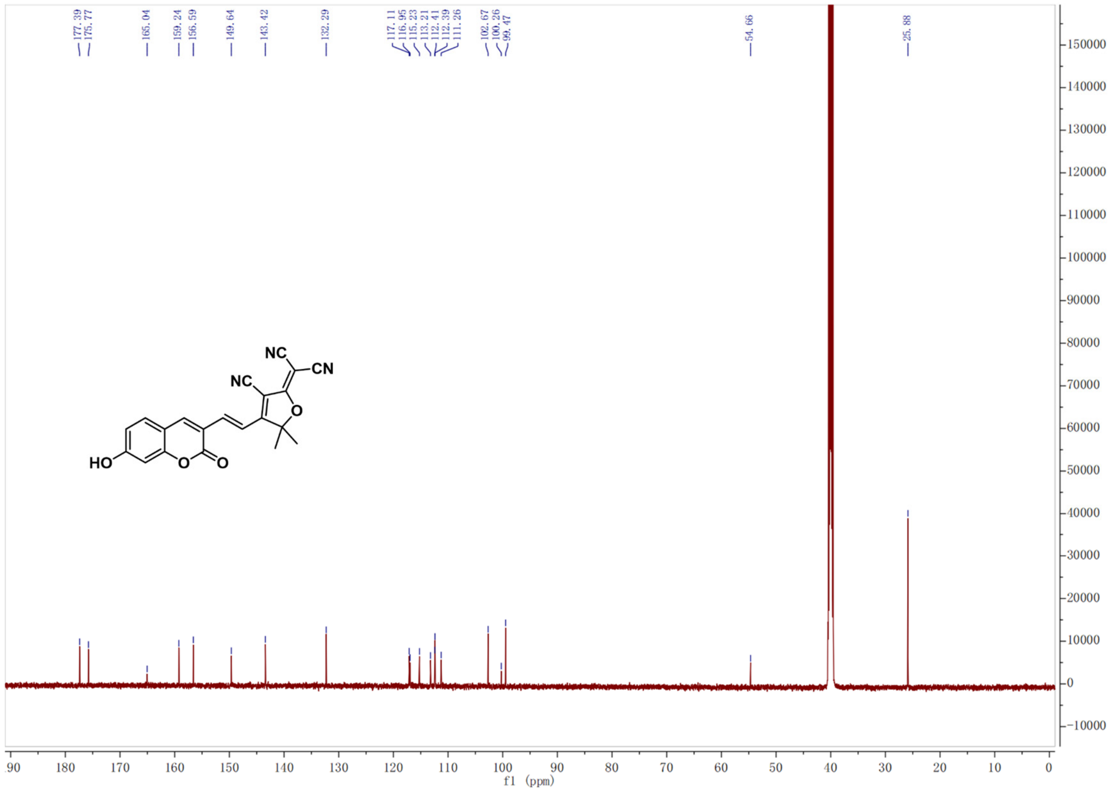

**Figure S10.** $^{13}C$ NMR spectrum of fluorophore **E**.

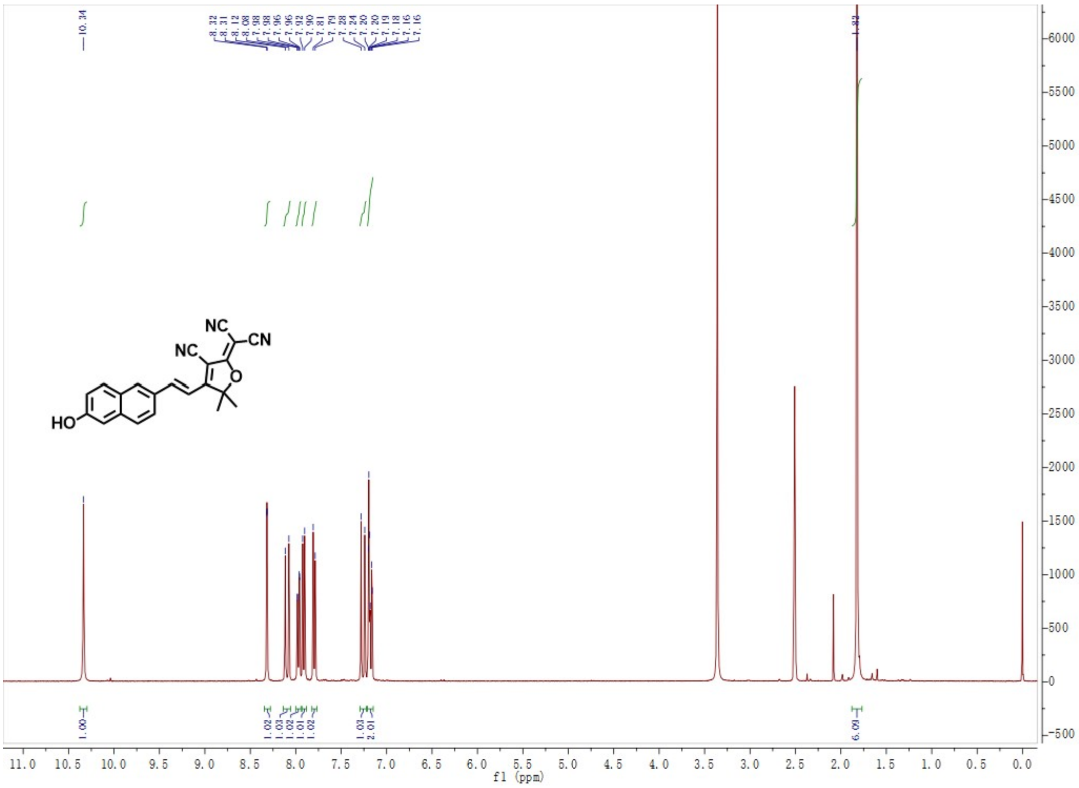


**Figure S11.** $^{1}$H NMR spectrum of fluorophore **F**.

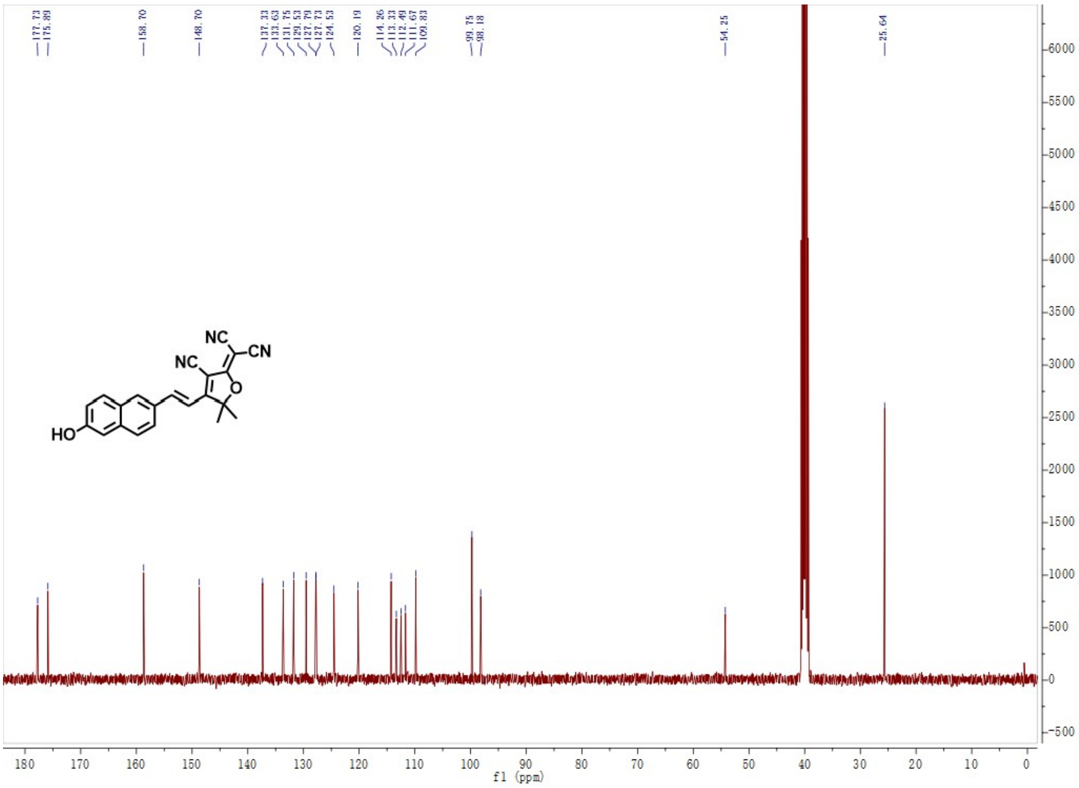


**Figure S12.** $^{13}$C NMR spectrum of fluorophore **F**.

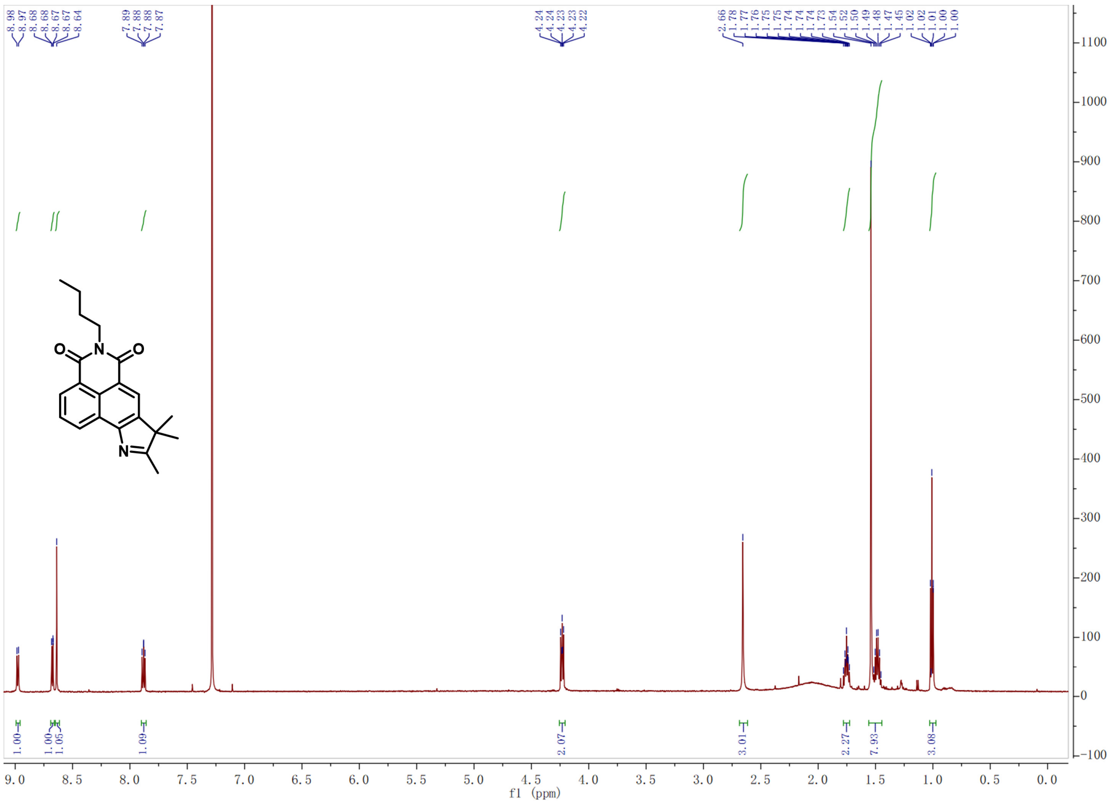

**Figure S13.** $^{1}$H NMR spectrum of **1-7**.

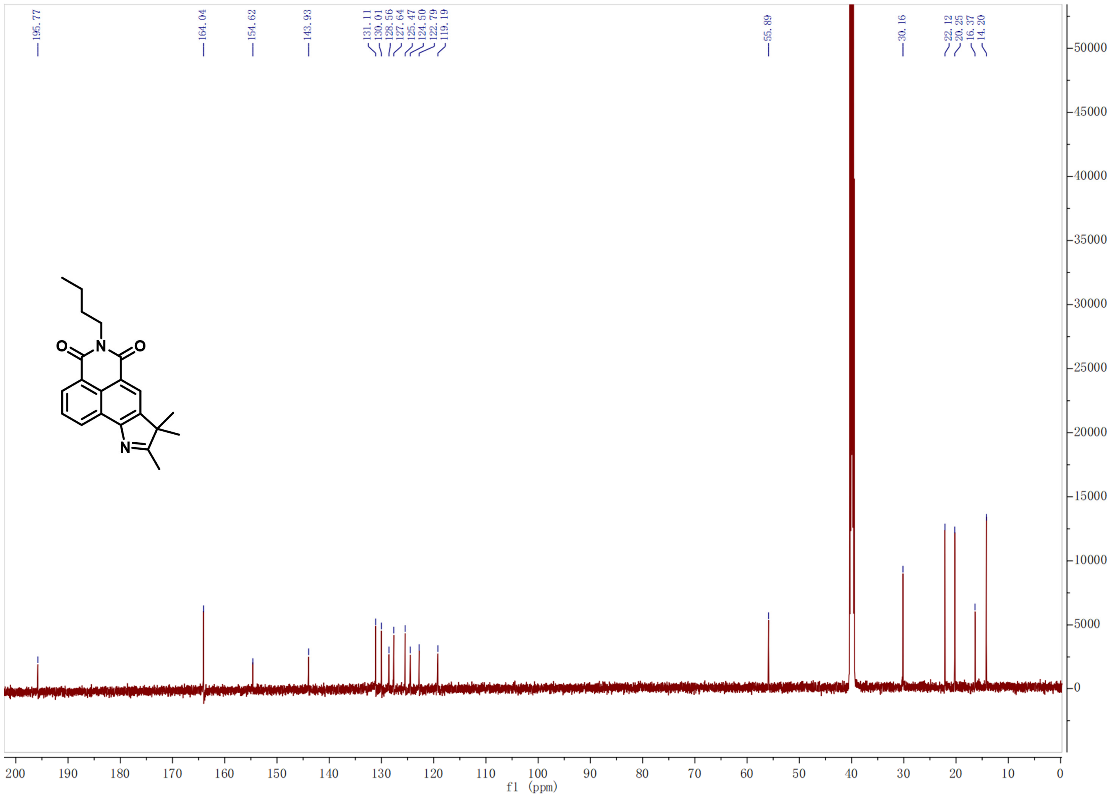

**Figure S14.** $^{13}$C NMR spectrum of **1-7**.

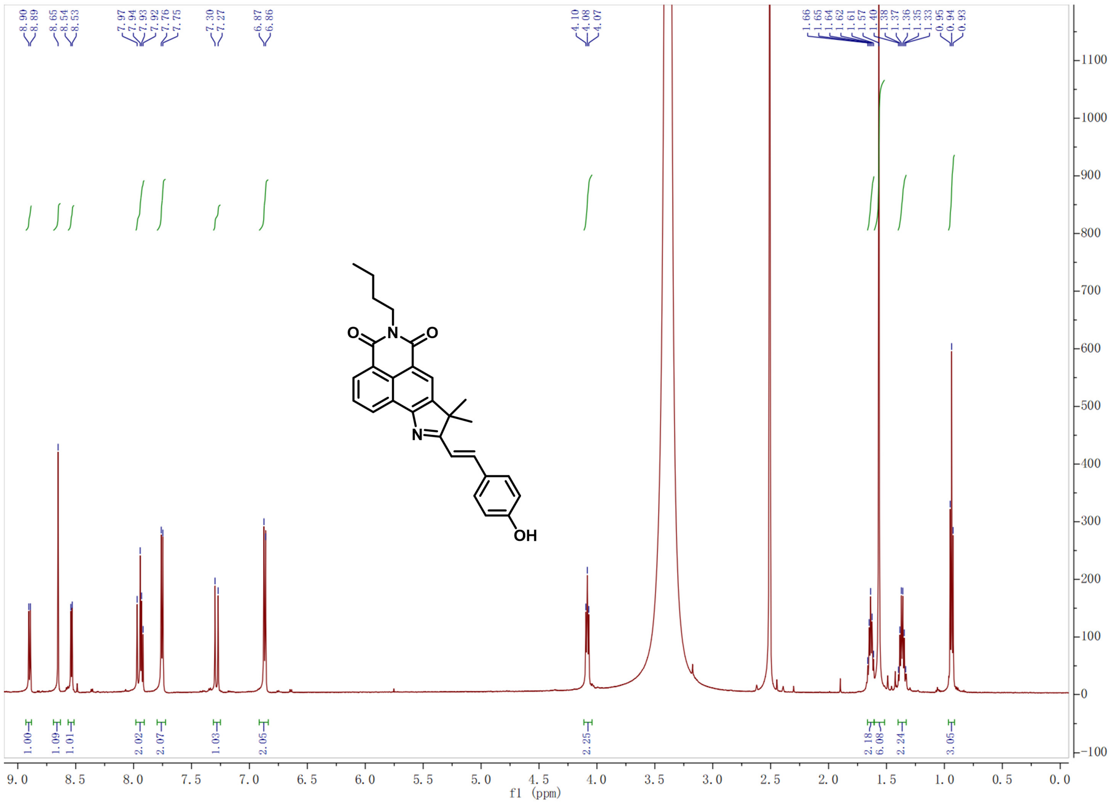

**Figure S15.** $^{1}H$ NMR spectrum of fluorophore **G**.

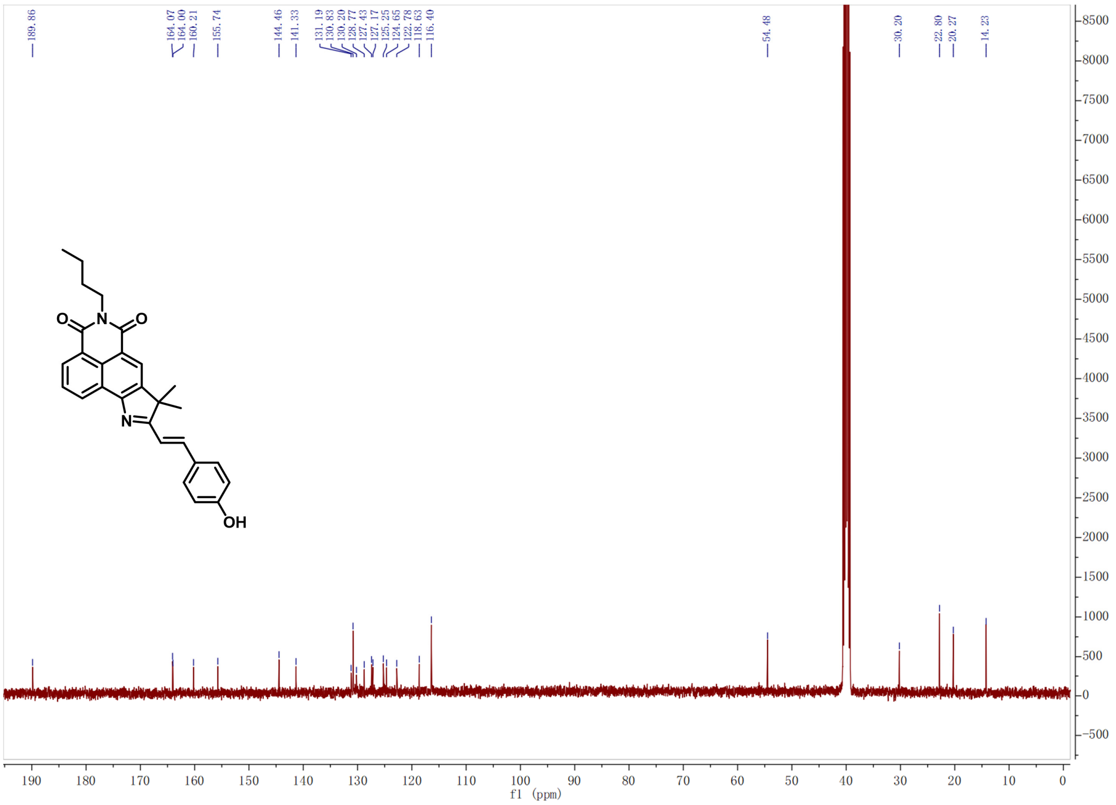

**Figure S16.** $^{13}C$ NMR spectrum of fluorophore **G**.

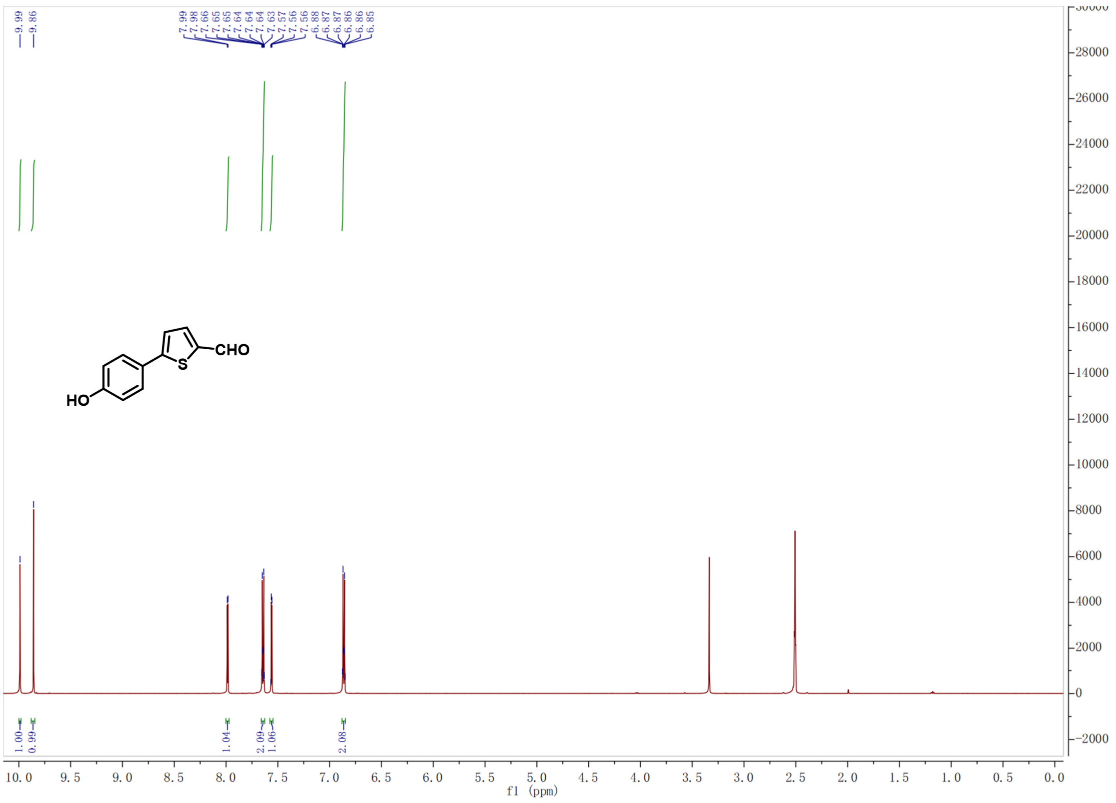


**Figure S17.** $^{1}H$ NMR spectrum of **1-8**.

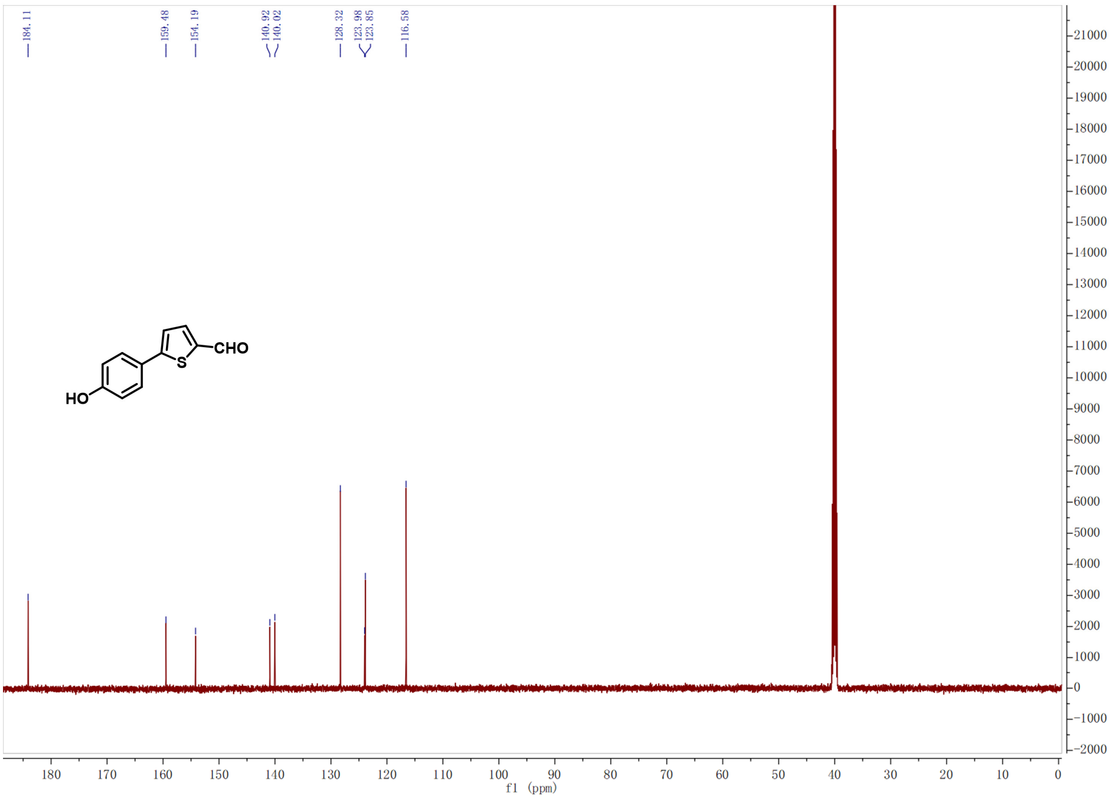


**Figure S18.** $^{13}C$ NMR spectrum of **1-8**.

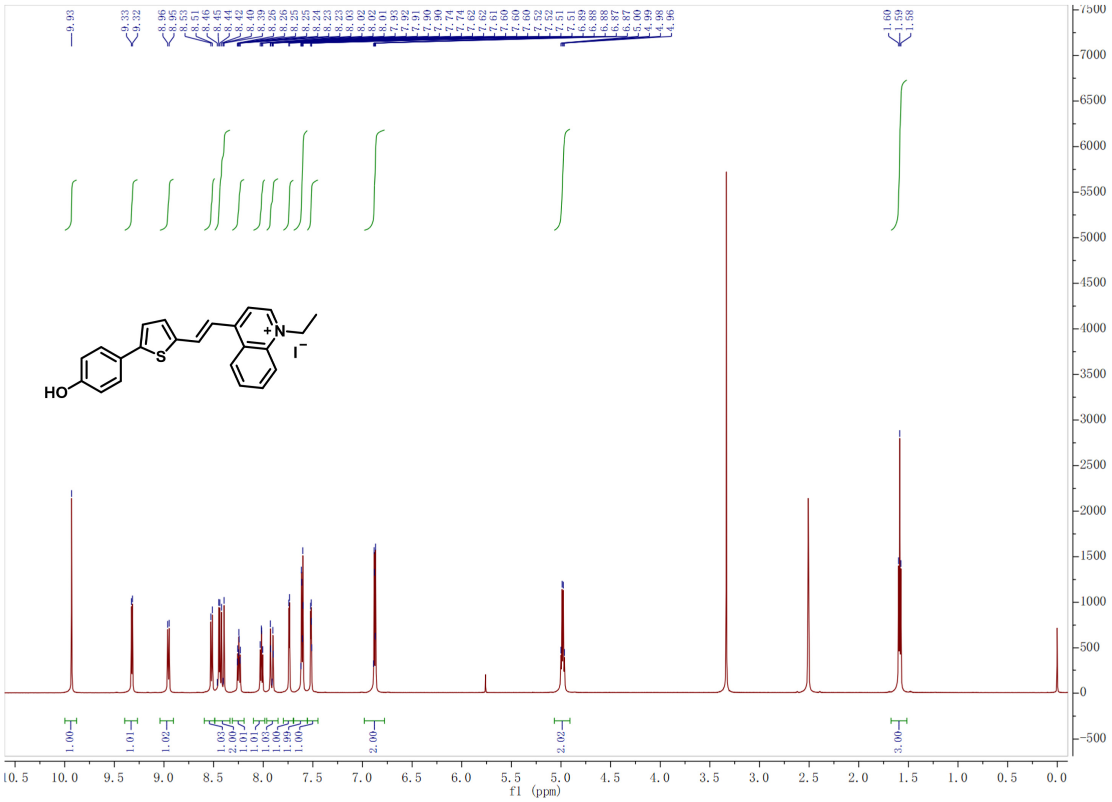

**Figure S19.** $^{1}$H NMR spectrum of fluorophore **H**.

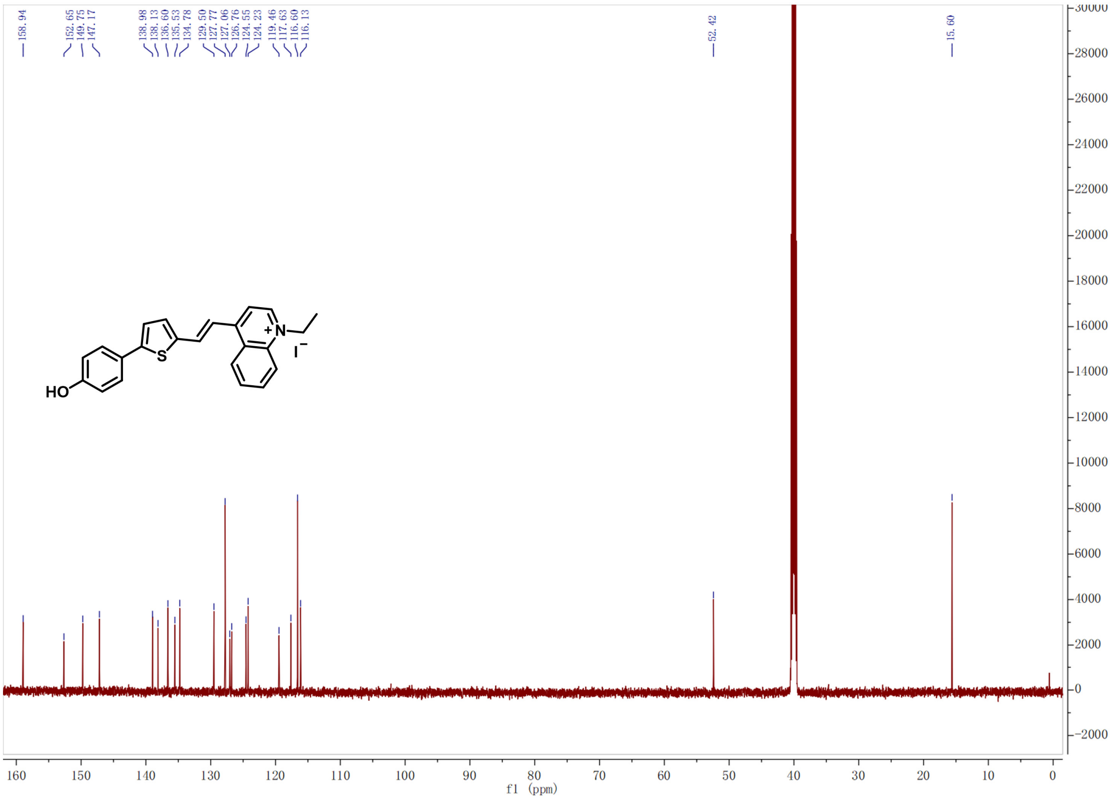

**Figure S20.** $^{13}$C NMR spectrum of fluorophore **H**.

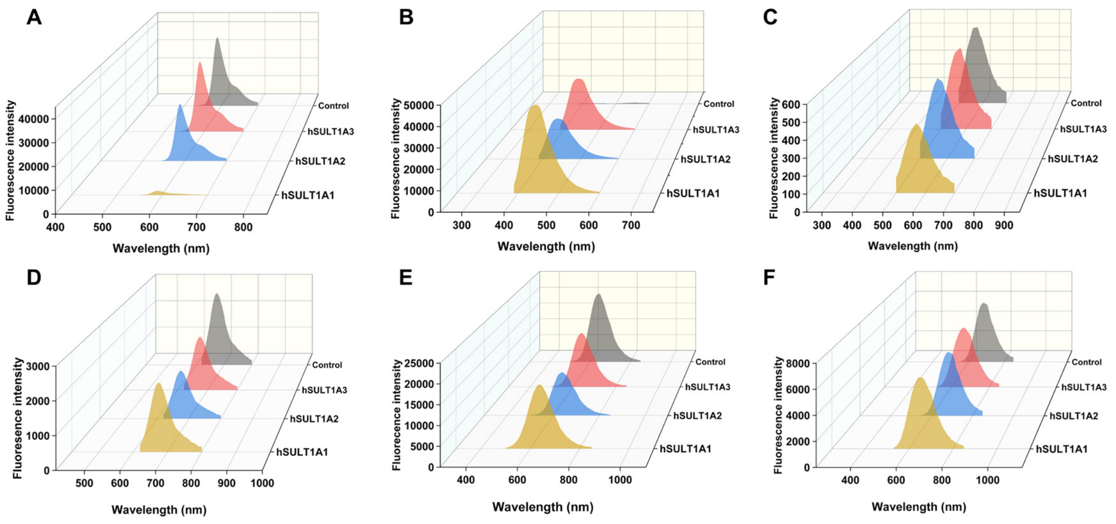


**Figure S21**. The emission spectra of fluorophore **A**, **B**, **D**, **E**, **F**, and **H** catalyzed by hSULT1As.

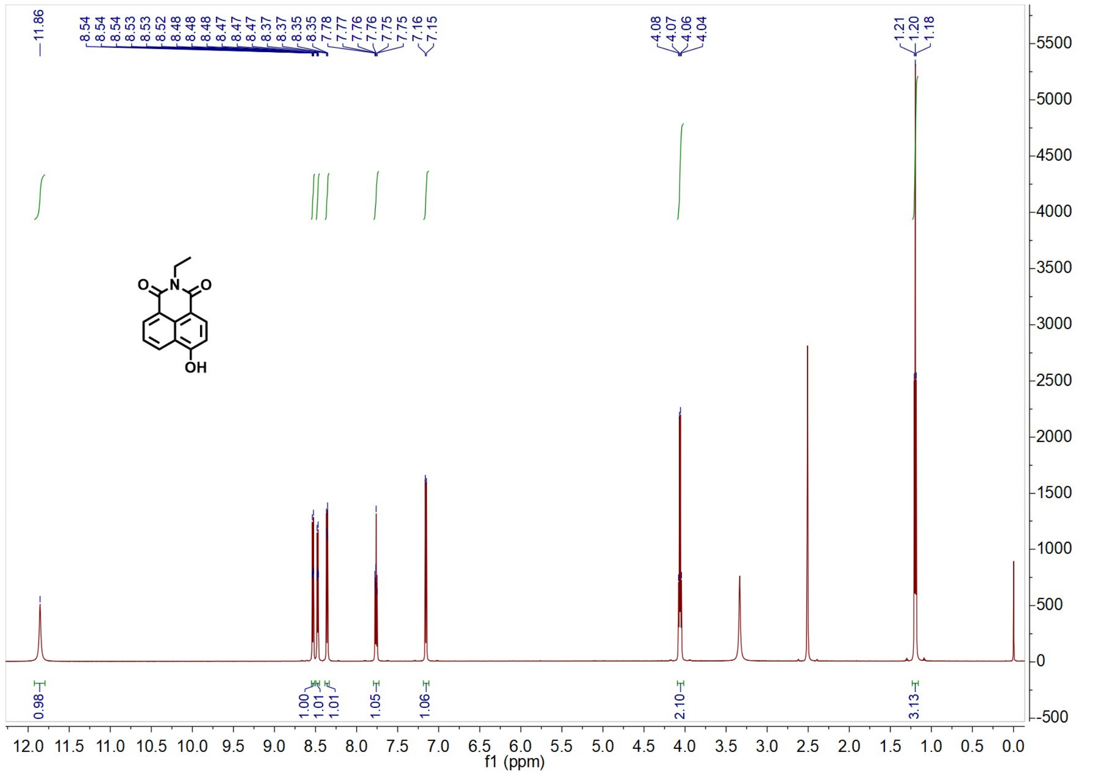


**Figure S22.** $^{1}$H NMR spectrum of **HN-241**.

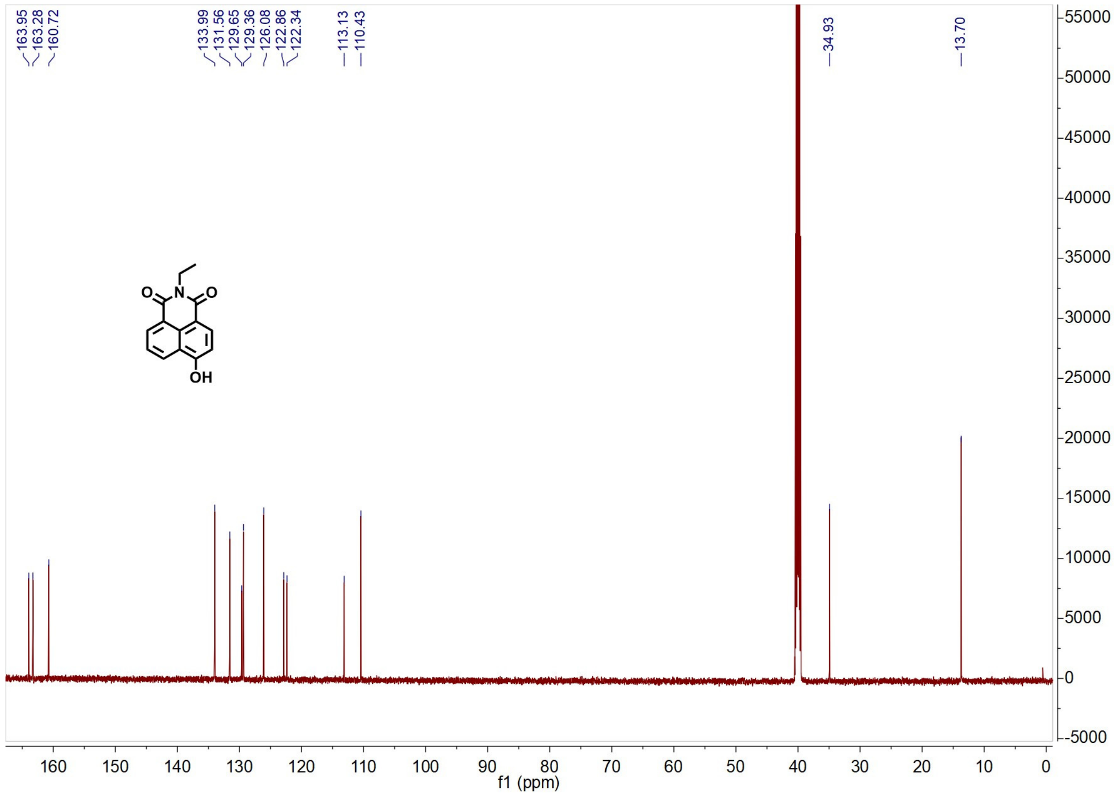


**Figure S23.** $^{13}$C NMR spectrum of **HN-241**.

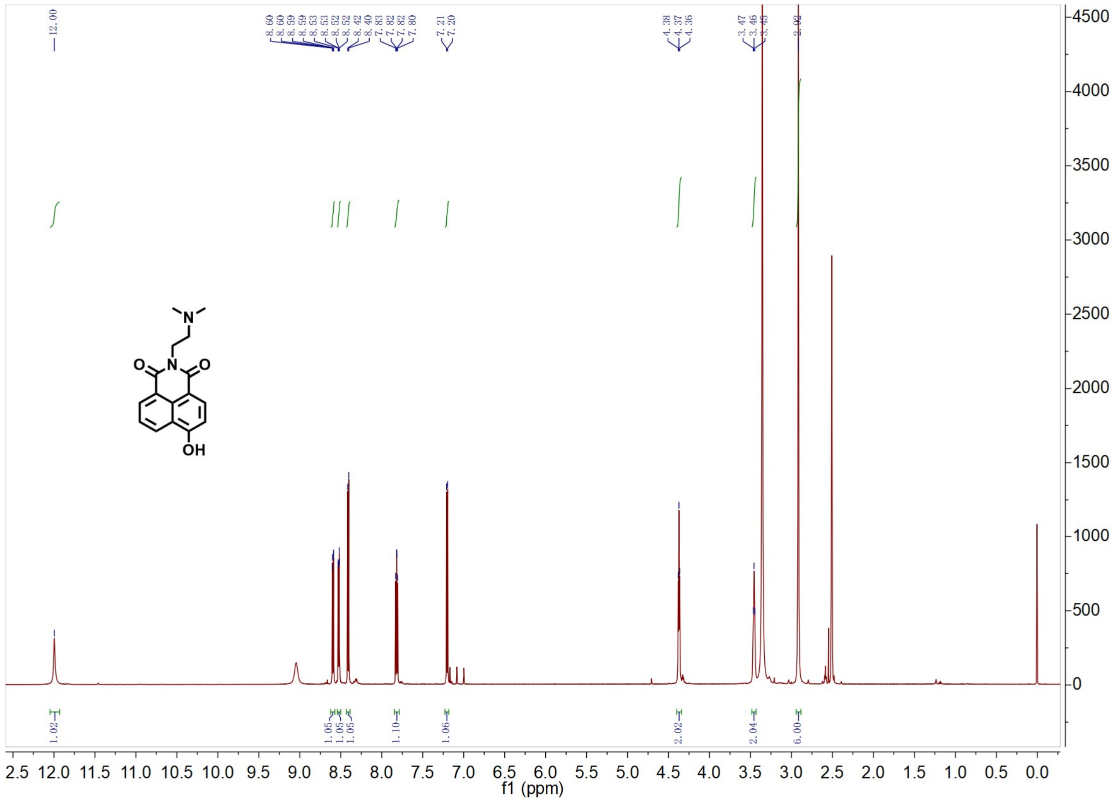


**Figure S24.** $^{1}$H NMR spectrum of **HN-283**.

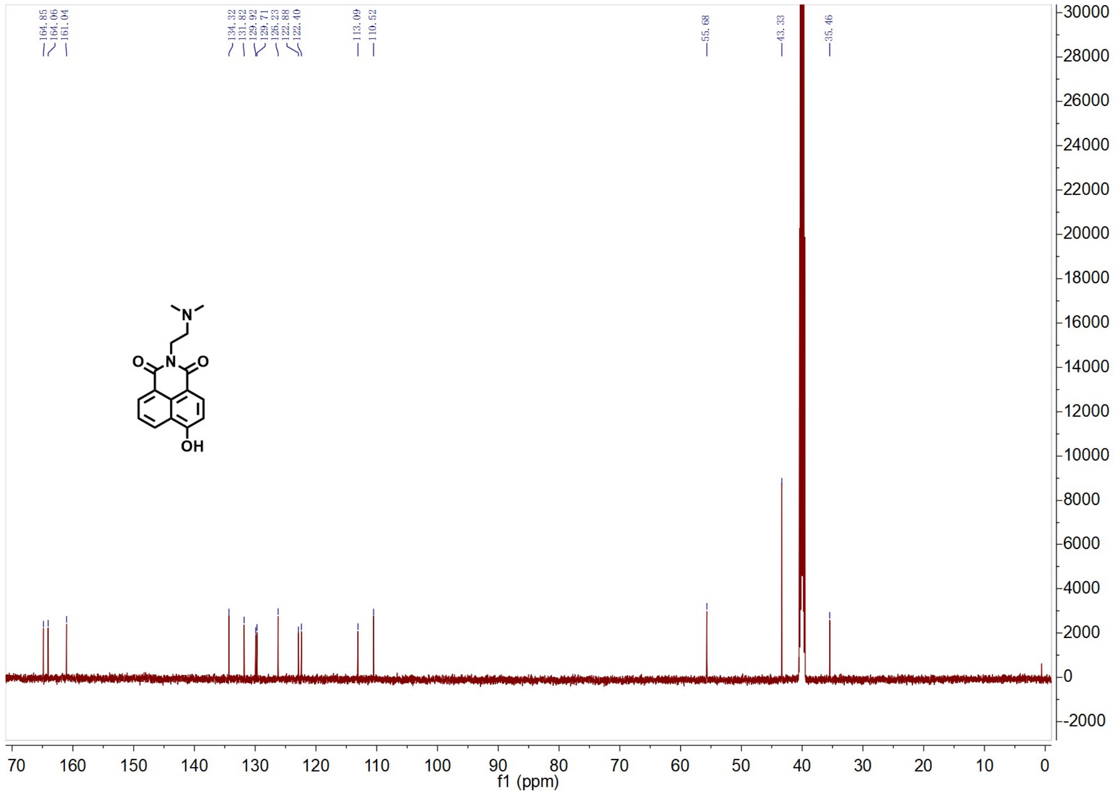


**Figure S25.** $^{13}C$ NMR spectrum of **HN-283**.

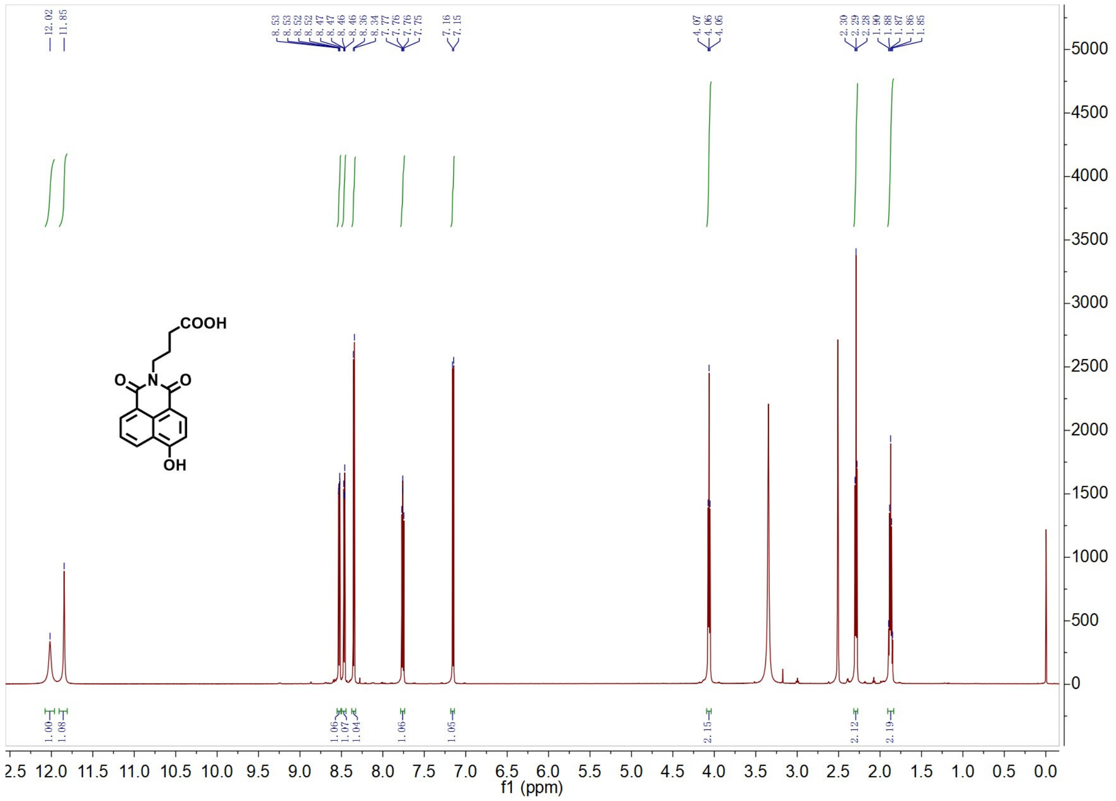


**Figure S26.** $^{1}H$ NMR spectrum of **HN-299**.

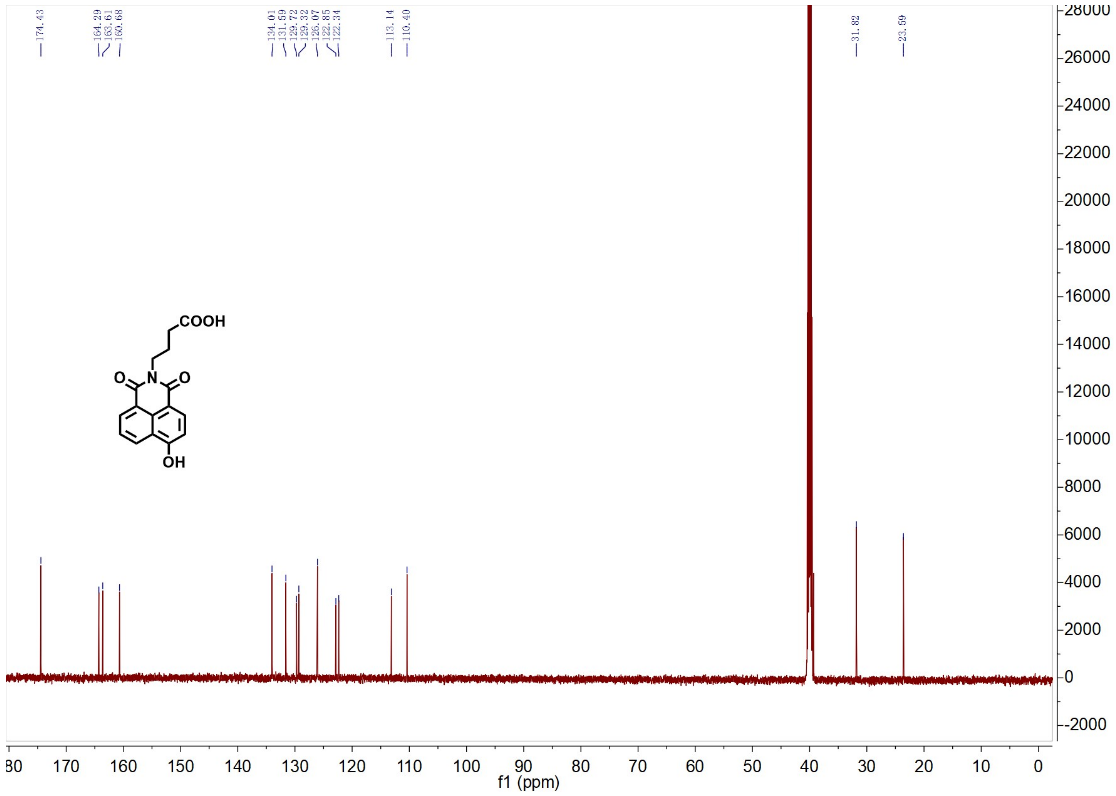


**Figure S27.** $^{13}$C NMR spectrum of **HN-299**.

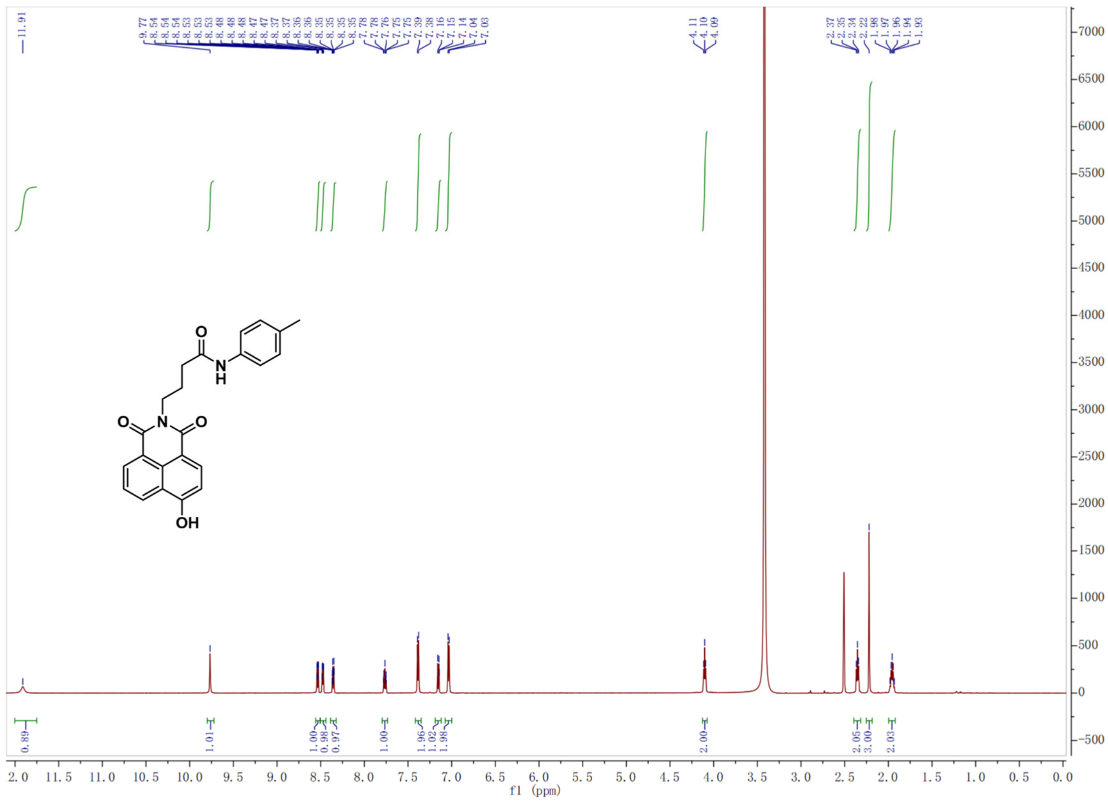


**Figure S28.** $^{1}$H NMR spectrum of **HN-388**.

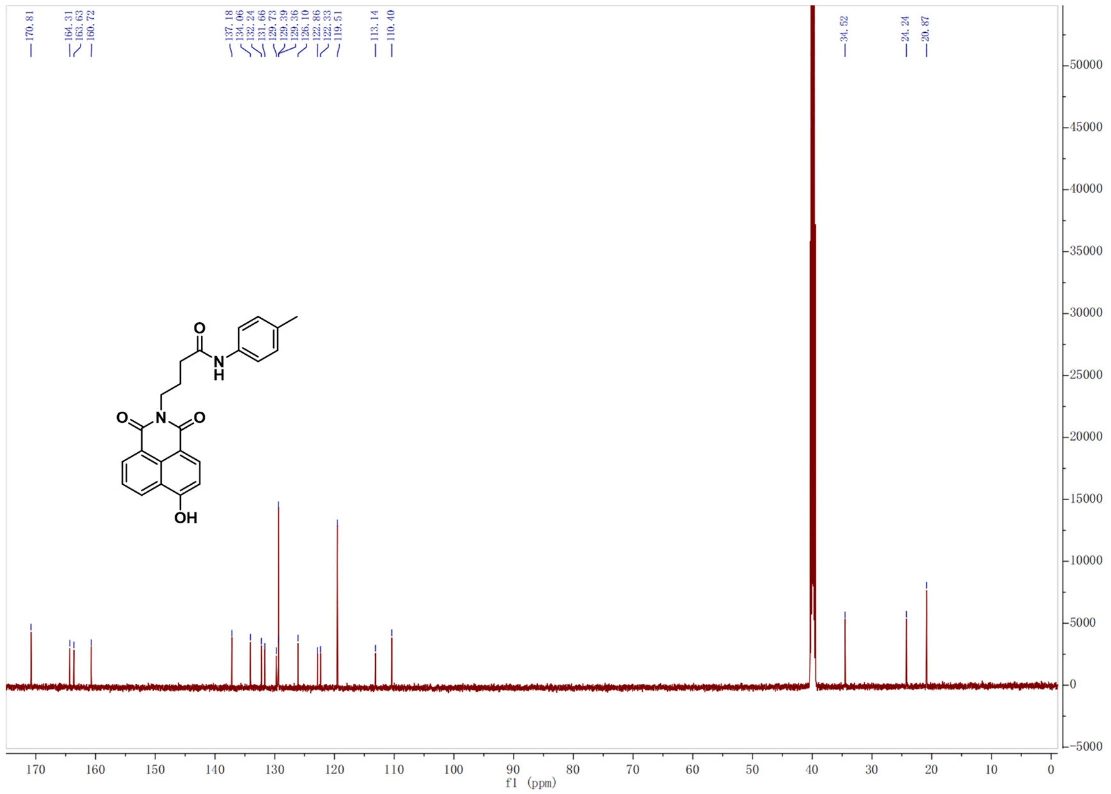

**Figure S29.** $^{13}C$ NMR spectrum of **HN-388**.

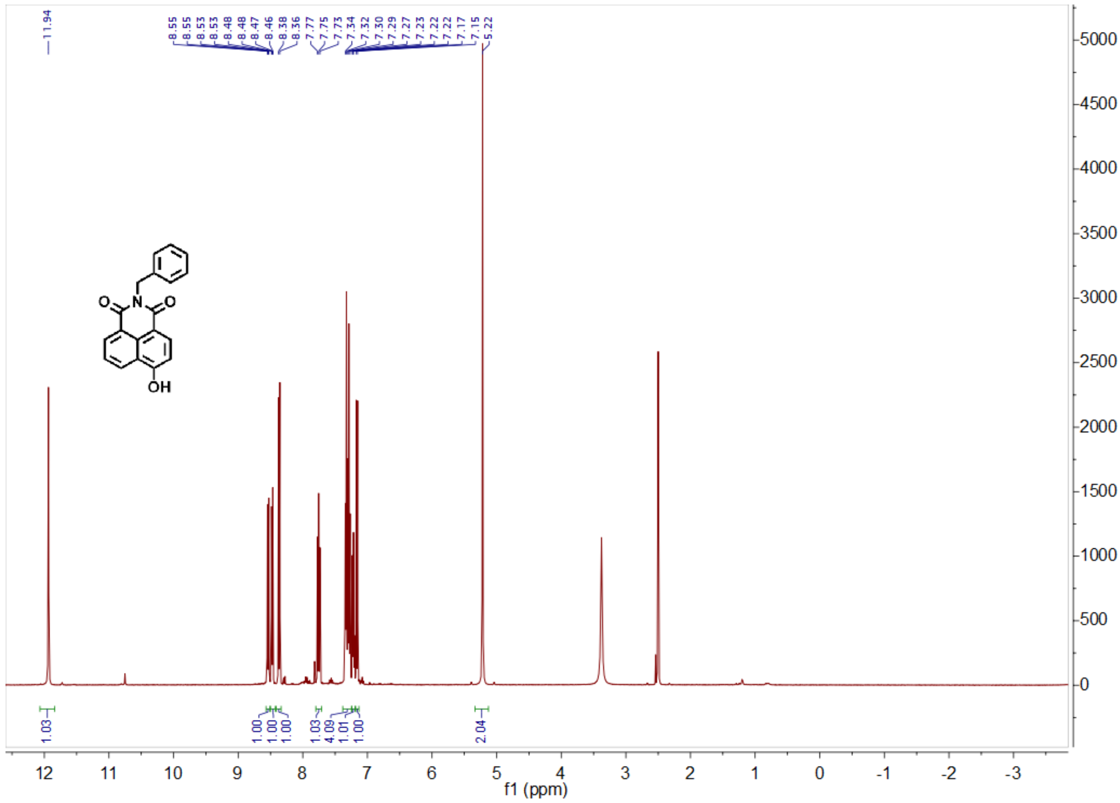

**Figure S30.** $^{1}H$ NMR spectrum of **HN-303**.

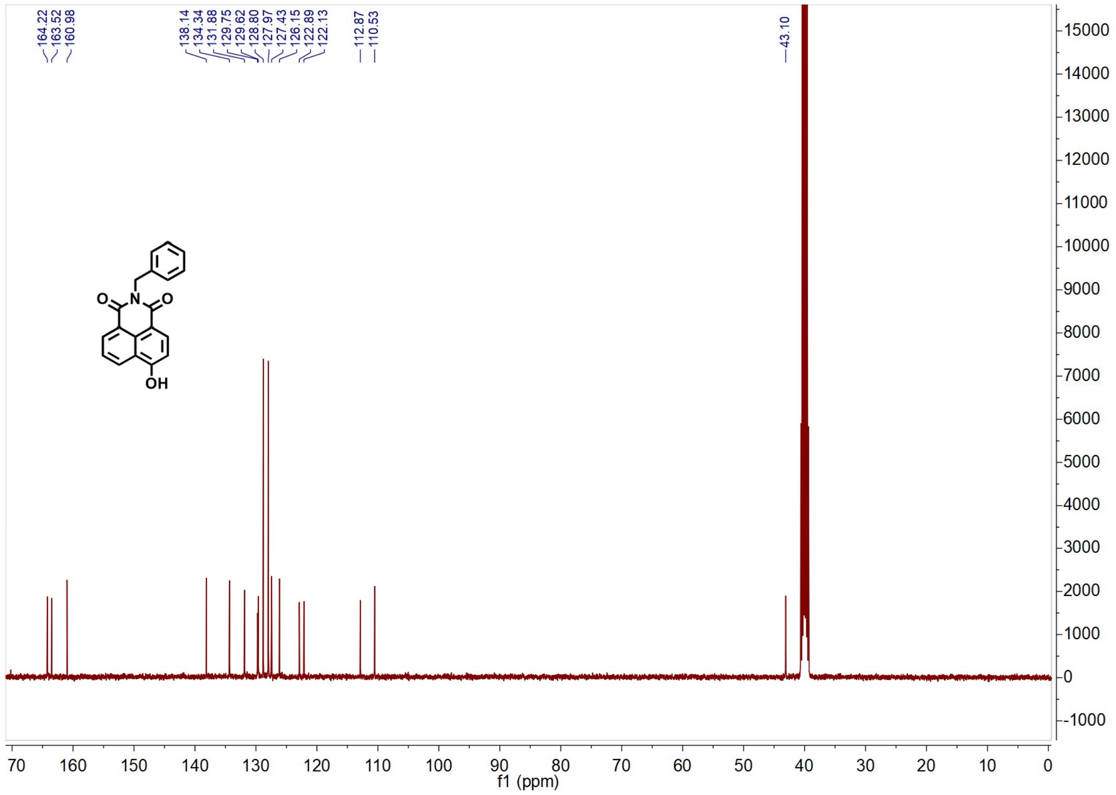


**Figure S31.** $^{13}$C NMR spectrum of **HN-303**.

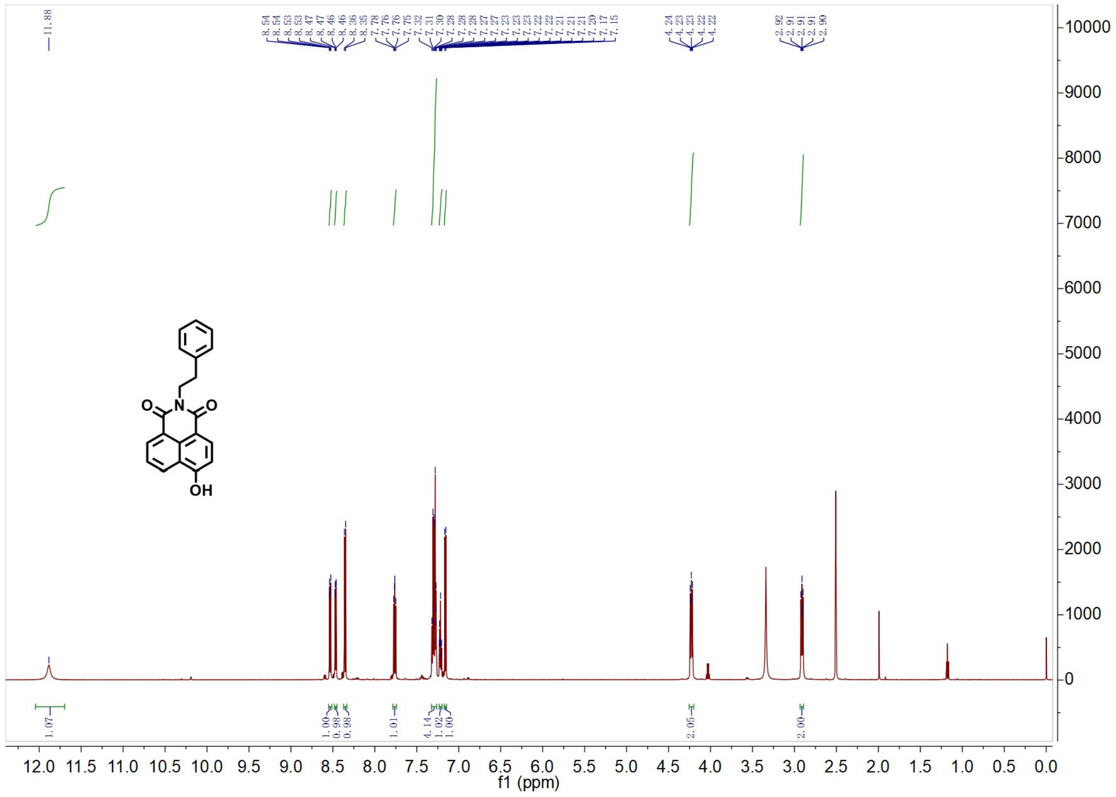


**Figure S32.** $^{1}$H NMR spectrum of **HN-317**.

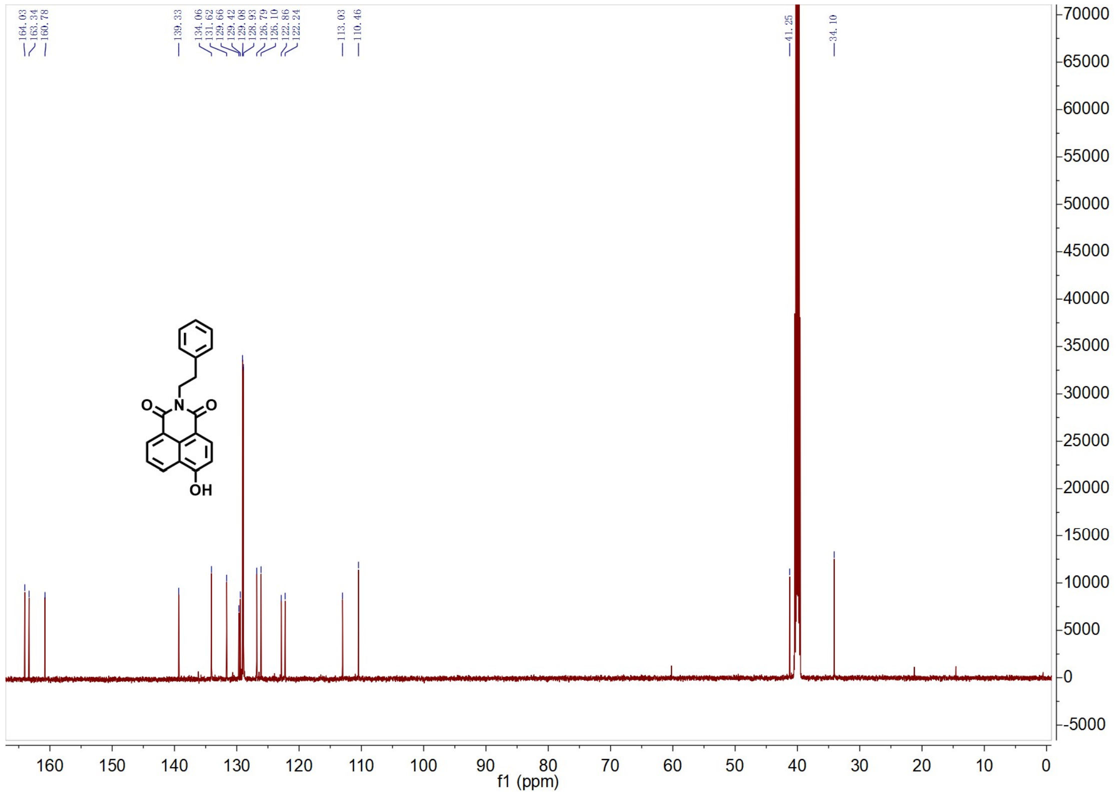

**Figure S33.** $^{13}$C NMR spectrum of **HN-317**.

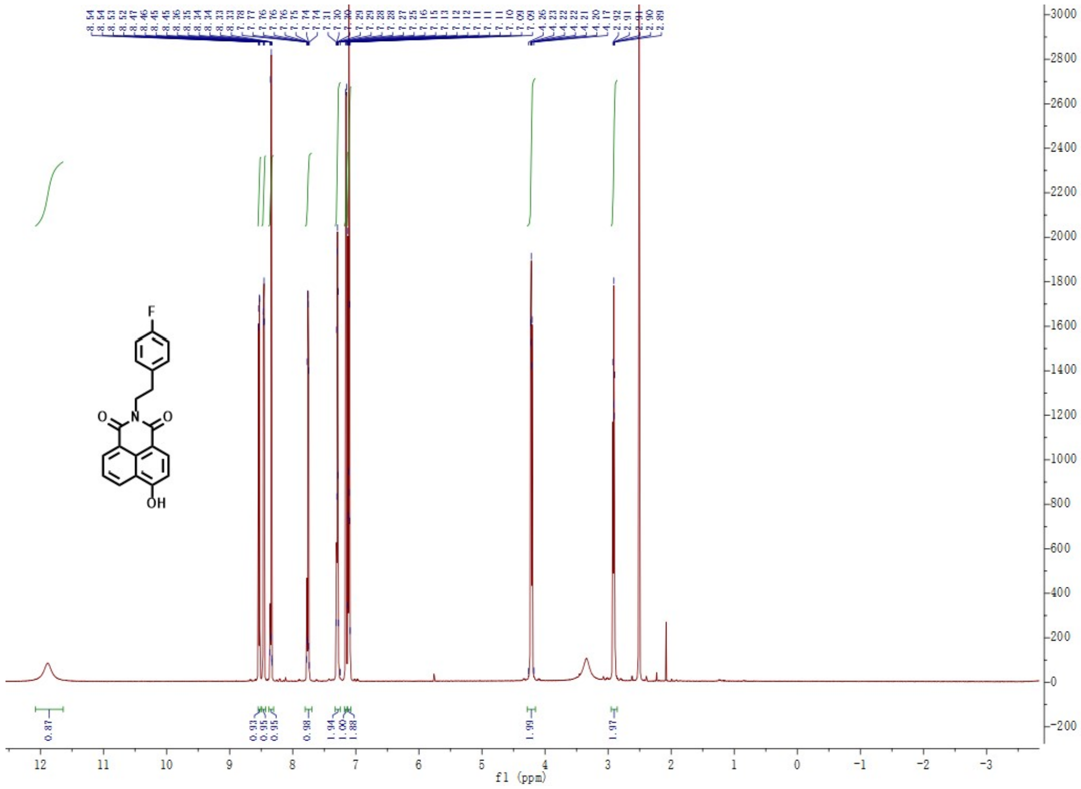

**Figure S34.** $^{1}$H NMR spectrum of **HN-335**.

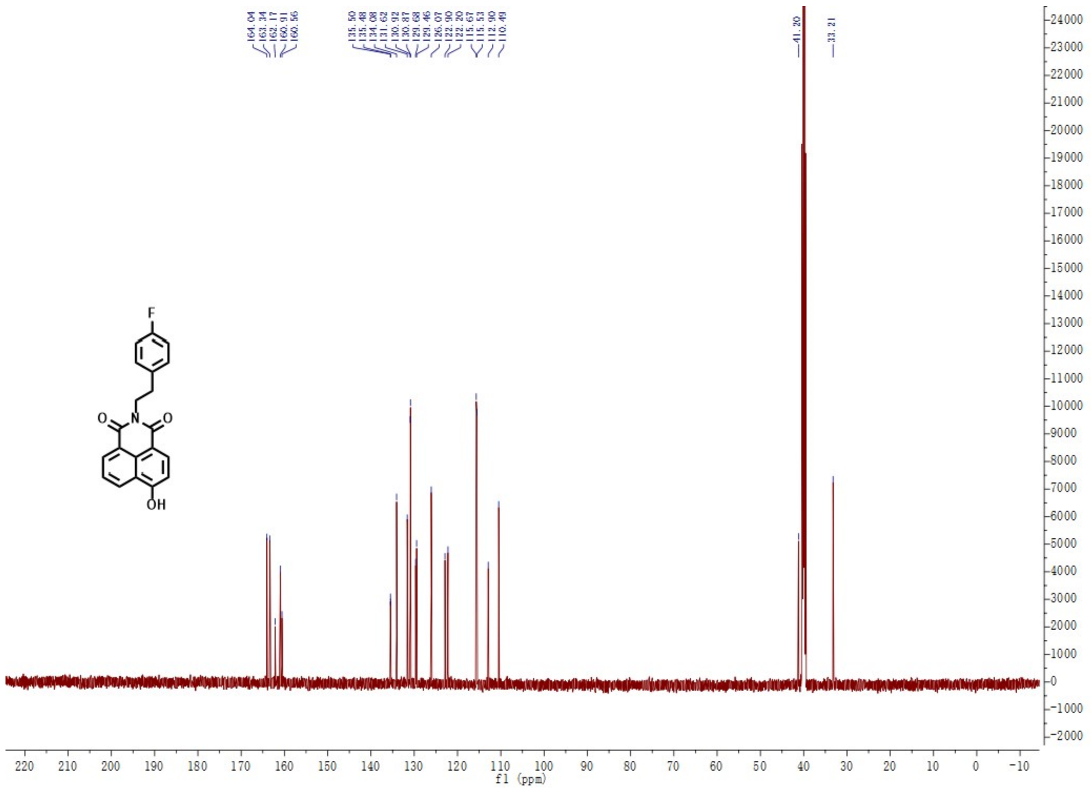

**Figure S35.** $^{13}$C NMR spectrum of **HN-335**.

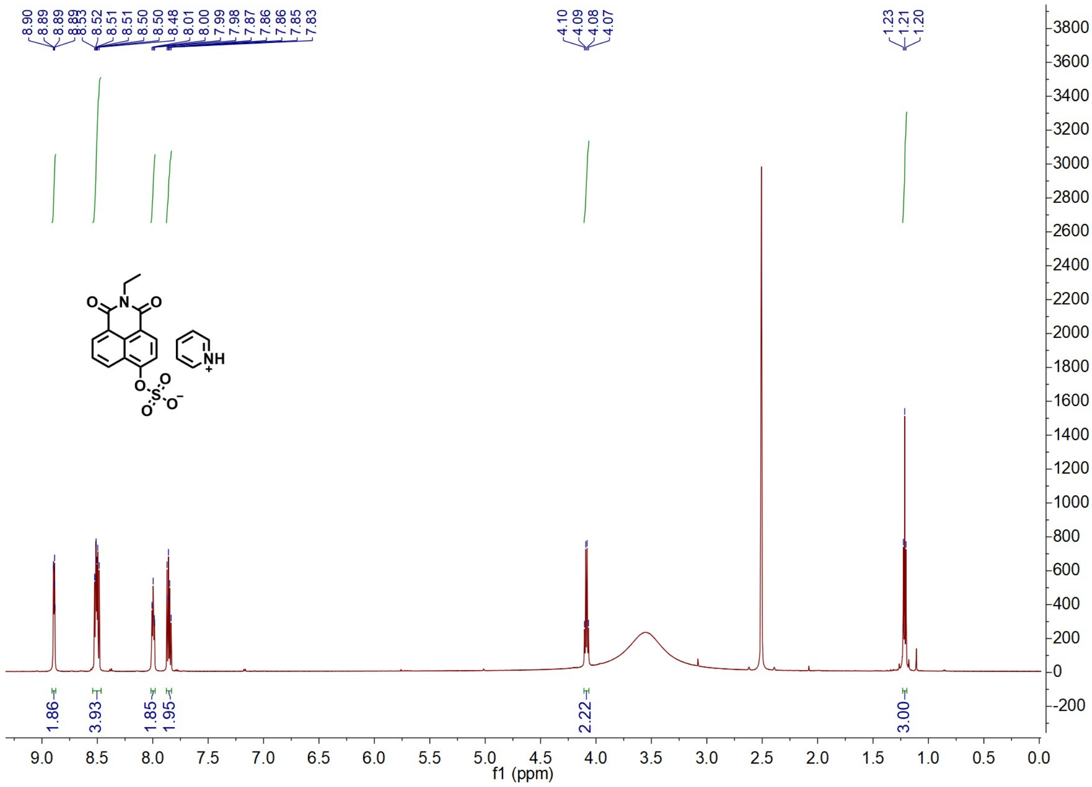

**Figure S36.** $^{1}$H NMR spectrum of **HN-241 sulfate**.

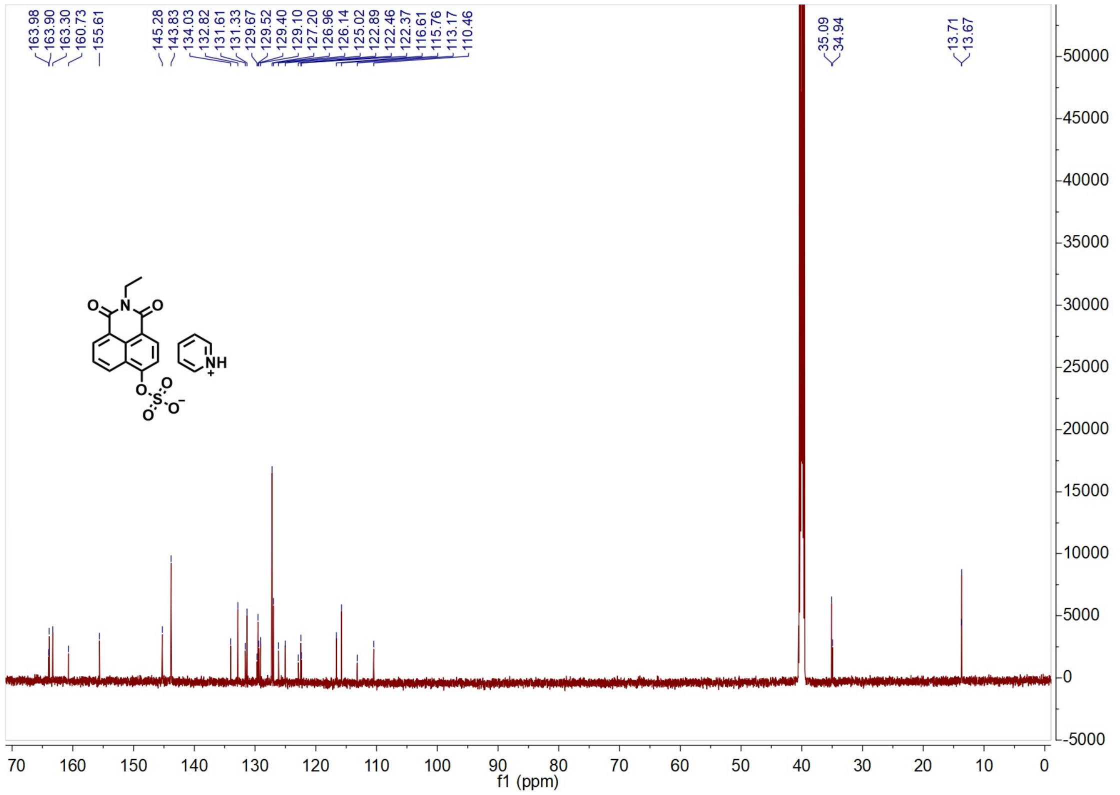

**Figure S37.** $^{13}$C NMR spectrum of **HN-241 sulfate**.

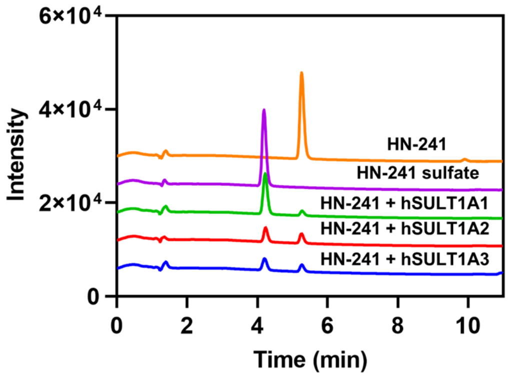


**Figure S38**. The HPLC profiles of **HN-241** and its sulfation metabolite (**HN-241 sulfate**).

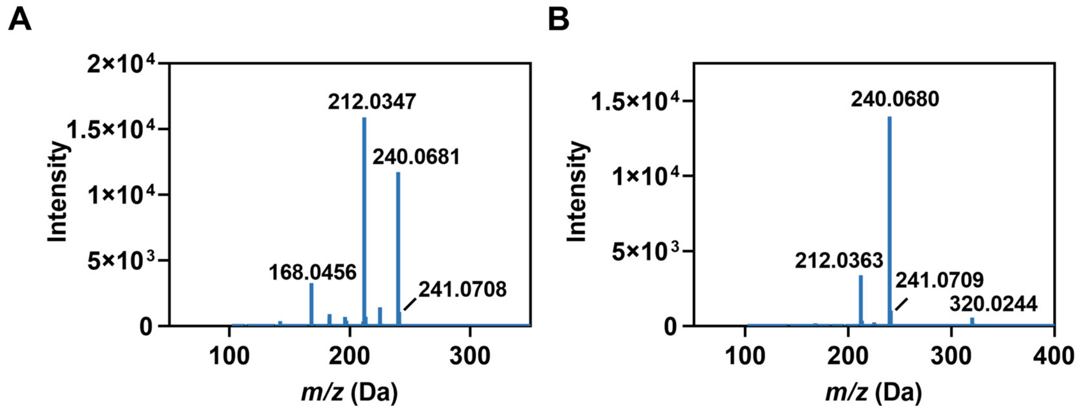

**Figure S39**. The MS/MS spectra of **HN-241** and its sulfation metabolite (**HN-241 sulfate**).

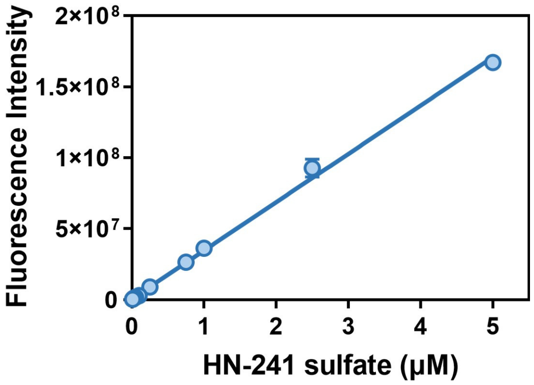


**Figure S40**. The standard curve of **HN-241 sulfate** using LC-FD.

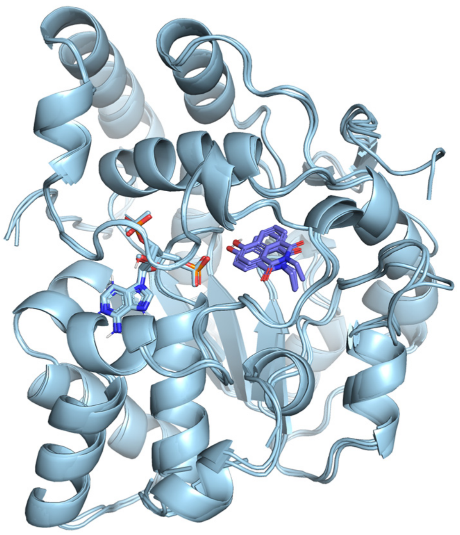

**Figure S41**. The molecular docking of **HN-241** (deep bule) in hSULT1A1, hSULT1A2, and hSULT1A3.

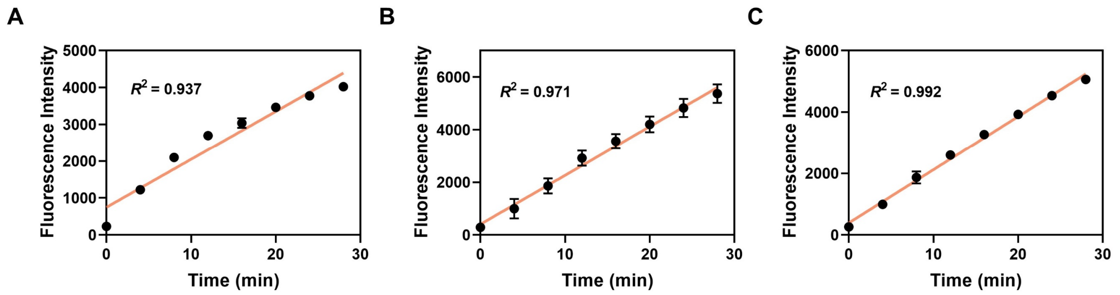


**Figure S42**. The liner relationship of fluorescence intensity at 450 nm for different reaction times (0–30 min) for hSULT1A1 (A), hSULT1A2 (B), and hSULT1A3 (C) catalyzed **HN-241** 4-*O*-sulfation.

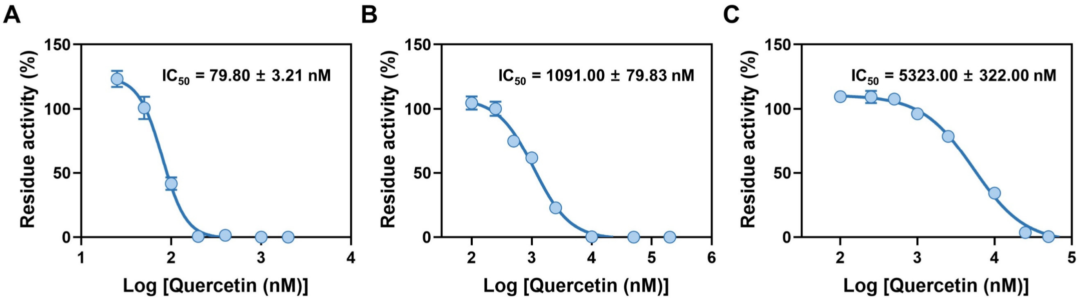


**Figure S43**. Dose-inhibition curves of quercetin against hSULT1A1 (A), hSULT1A2 (B), hSULT1A3-catalyzed (C) **HN-241** 4-*O*-sulfation.

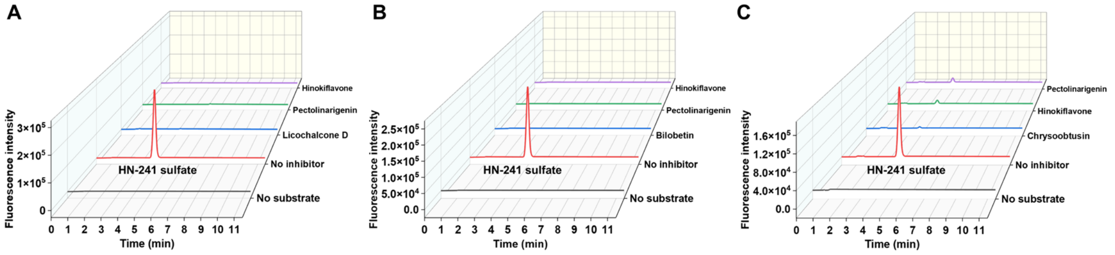


**Figure S44**. (A) The chromatograms of **HN-241** in the presence of PAPS and hSULT1A1 with or without the newly identified hSULT1A1 inhibitor. (B) The chromatograms of **HN-241** in the presence of PAPS and hSULT1A2 with or without the newly identified hSULT1A2 inhibitor. (C) The chromatograms of **HN-241** in the presence of PAPS and hSULT1A3 with or without the newly identified hSULT1A3 inhibitor. **HN-241 sulfate** detected by LC-FD with excitation wavelength ($\lambda_{ex}$) and emission wavelength ($\lambda_{em}$) at 350 nm and 450 nm.

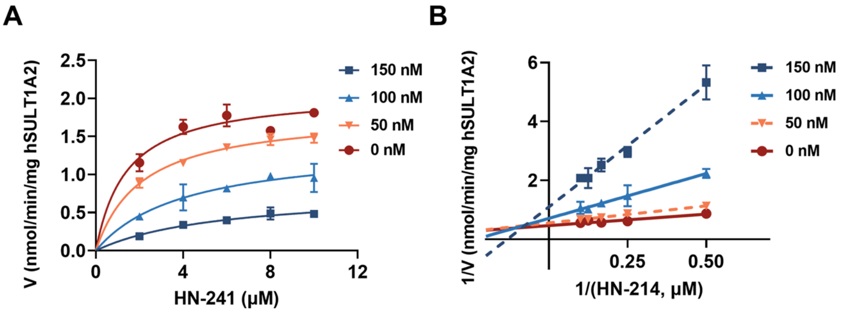


**Figure S45**. (A) Inhibition kinetics of bilobetin against hSULT1A2. (B) The second plot of slope from the Lineweaver-Burk plot for inhibition of bilobetin against hSULT1A2.

**Table S1**. The molecular docking of **HN-241** in hSULT1As.

| Enzyme | Affinity (kcal/mol) | Conformations[1] |
|---|---|---|
| hSULT1A1 | –8.3 | 4 |
| hSULT1A2 | –7.1 | 5 |
| hSULT1A3 | –7.1 | 3 |

[1] The formed catalyzed conformation defined as the distance between Nε2 in His-108 of hSULT1As and the oxygen atom in the hydroxyl group of **HN-241** less than 5 Å.

**Table S2**. The reaction conditions of screening system for hSULT1As inhibitors.

| Enzyme | Substrate concentration (μM) | Enzyme concentration (pmol) | Reaction time (min) |
|---|---|---|---|
| hSULT1A1 | 1 | 6 | 15 |
| hSULT1A2 | 5 | 29 | 15 |
| hSULT1A3 | 10 | 29 | 15 |

**Table S3**. Inhibitory activity of 94 natural compounds against hSULT1As-catalyzed **HN-241** 4-*O*-sulfation.

| No. | Compound | CAS number | Molecular weight | Residue activity (%) | | |
|---|---|---|---|---|---|---|
| | | | | hSULT1A1 | hSULT1A2 | hSULT1A3 |
| A1 | Podophyllotoxin | 518-28-5 | 414.41 | 110.34 | 88.89 | 110.88 |
| A2 | Alpinetin | 36052-37-6 | 270.28 | 69.79 | 49.53 | 115.04 |
| A3 | Taxifolin | 98006-93-0 | 304.25 | 3.26 | 41.73 | 59.70 |
| A4 | Crotaline | 315-22-0 | 325.36 | 128.03 | 97.30 | 122.18 |
| A5 | Sciadopitysin | 521-34-6 | 580.54 | 27.69 | 2.03 | 62.24 |
| A6 | Schisandrin | 7432-28-2 | 432.51 | 129.29 | 102.92 | 123.25 |
| A7 | Methyl caffeate acid | 3843-74-1 | 194.18 | 9.01 | 12.99 | 44.99 |
| A8 | Loganic acid | 22255-40-9 | 376.36 | 127.71 | 98.56 | 118.96 |
| A9 | Narcissoside | 604-80-8 | 624.54 | 99.20 | 86.78 | 60.08 |
| A10 | Gomisin A | 58546-54-6 | 416.46 | 129.03 | 97.44 | 122.78 |
| A11 | Shikonin | 517-89-5 | 288.30 | 64.52 | 18.60 | 60.98 |
| A12 | Rhein | 478-43-3 | 284.22 | 59.39 | 0.83 | 96.92 |
| B1 | Chrysophanic acid | 481-74-3 | 254.24 | 3.52 | 45.37 | 99.34 |
| B2 | 4-Methoxychalcone | 959-33-1 | 238.28 | 78.44 | 75.95 | 111.48 |
| B3 | Loureirin B | 119425-90-0 | 316.35 | 5.84 | 6.16 | 71.91 |
| B4 | Resibufogenin | 465-39-4 | 384.51 | 126.93 | 99.24 | 112.79 |
| B5 | Ginkgolic acid | 111047-30-4 | 374.56 | 12.05 | 13.44 | 66.66 |

| | | | | | | |
|---|---|---|---|---|---|---|
| B6 | 3β,5,14-Trihydroxy-5β-bufa-20,22-dienolide | 472-26-4 | 402.52 | 121.35 | 109.91 | 114.49 |
| B7 | Oleanolic acid | 508-02-1 | 456.70 | 126.78 | 101.29 | 114.45 |
| B8 | Myricetin | 529-44-2 | 318.24 | 0.08 | 4.43 | 39.81 |
| B9 | Ammothamnine | 16837-52-8 | 264.36 | 99.18 | 96.56 | 100.34 |
| B10 | Emodin-3-methyl ether | 521-61-9 | 284.26 | 54.41 | 89.81 | 103.18 |
| B11 | Alnustone | 33457-62-4 | 262.35 | 74.39 | 80.60 | 111.42 |
| B12 | 1,7-Dihydroxy-2,3,8-trimethoxy-6-methylanthracene-9,10-dione | 70588-05-5 | 344.32 | 0.00 | 0.20 | 61.63 |
| C1 | Isoimperatorin | 482-45-1 | 270.28 | 92.52 | 106.05 | 92.11 |
| C2 | Chrysoobtusin | 70588-06-6 | 358.34 | 1.84 | 0.09 | 5.28 |
| C3 | Isorhamnetin | 480-19-3 | 316.26 | 1.36 | 0.39 | 58.31 |
| C4 | Glycitin | 40246-10-4 | 446.41 | 98.37 | 97.40 | 114.83 |
| C5 | Sennosides | 517-43-1 | 862.74 | 26.92 | 3.06 | 19.21 |
| C6 | Worenine | 38763-29-0 | 334.35 | 101.91 | 92.89 | 125.56 |
| C7 | Chenodeoxycholic acid | 474-25-9 | 392.57 | 96.52 | 98.24 | 129.10 |
| C8 | Aurantio-obtusin | 67979-25-3 | 330.29 | 0.45 | 0.00 | 45.21 |
| C9 | Imperatorin | 482-44-0 | 270.28 | 100.94 | 94.32 | 128.29 |
| C10 | trans-Chalcone | 614-47-1 | 208.26 | 71.86 | 91.40 | 116.88 |
| C11 | Acacetin | 480-44-4 | 284.26 | 0.38 | 0.47 | 58.10 |
| C12 | Liquiritigenin | 578-86-9 | 256.25 | 58.91 | 25.92 | 76.68 |
| D1 | Baicalein | 491-67-8 | 270.24 | 0.00 | 0.02 | 8.08 |
| D2 | Licochalcone D | 144506-15-0 | 354.40 | 0.00 | 0.21 | 30.87 |
| D3 | Cardamonin | 19309-14-9 | 270.28 | 63.24 | 34.41 | 120.65 |
| D4 | Bufalin | 465-21-4 | 386.52 | 103.21 | 106.43 | 124.18 |
| D5 | Sinomenine | 115-53-7 | 329.39 | 103.39 | 103.38 | 110.25 |
| D6 | Icariside I | 56725-99-6 | 530.52 | 91.81 | 103.62 | 104.97 |
| D7 | Vitexin | 3681-93-4 | 432.38 | 101.73 | 114.66 | 107.53 |
| D8 | Hinokiflavone | 19202-36-9 | 538.46 | 0.00 | 0.00 | 9.46 |
| D9 | Morin | 480-16-0 | 302.24 | 50.72 | 4.73 | 55.69 |
| D10 | 5-Hydroxyfisetin | 490-31-3 | 302.24 | 2.58 | 1.21 | 43.68 |
| D11 | iso-Licoflavonol | 94805-83-1 | 354.35 | 3.31 | 0.03 | 62.13 |
| D12 | Pectolinarigenin | 520-12-7 | 314.29 | 1.12 | 0.00 | 1.34 |
| E1 | Calycosin-7-*O*-β-D-glucoside | 20633-67-4 | 446.40 | 2.77 | 22.81 | 54.16 |
| E2 | Rotenone | 83-79-4 | 394.42 | 108.55 | 102.31 | 106.16 |
| E3 | Mulberrin | 62949-79-5 | 422.47 | 53.37 | 143.65 | 70.86 |
| E4 | Hydroxysafflor yellow A | 78281-02-4 | 612.53 | 97.09 | 100.46 | 99.76 |
| E5 | (20R)-Protopanaxdiol | 7755-01-3 | 460.73 | 99.25 | 97.81 | 110.20 |
| E6 | Eriocitrin | 13463-28-0 | 596.53 | 65.53 | 110.95 | 104.32 |
| E7 | Zerumbone | 471-05-6 | 218.34 | 102.41 | 113.04 | 108.16 |
| E8 | 1,4-Anthracenedione | 635-12-1 | 208.21 | 29.15 | 1.06 | 94.23 |

| | | | | | | |
|---|---|---|---|---|---|---|
| E9 | Aloeemodin | 481-72-1 | 270.24 | 0.21 | 0.00 | 61.29 |
| E10 | Icariin | 489-32-7 | 676.66 | 85.55 | 85.06 | 104.78 |
| E11 | Neoisoliquiritigenin | 7014-39-3 | 418.39 | 91.36 | 84.48 | 108.00 |
| E12 | Emodin | 518-82-1 | 270.24 | 0.00 | 0.00 | 65.01 |
| F1 | Triptolide | 38748-32-2 | 360.40 | 109.34 | 119.12 | 109.92 |
| F2 | Mesaconitine | 2752-64-9 | 631.71 | 111.12 | 114.51 | 110.31 |
| F3 | Liquiritin | 551-15-5 | 418.39 | 100.00 | 95.10 | 100.00 |
| F4 | 20(S)-Ginsenoside Rh2 | 78214-33-2 | 622.87 | 85.94 | 110.74 | 105.44 |
| F5 | 20(R)-Ginsenoside Rh2 | 112246-15-8 | 622.88 | 98.01 | 114.69 | 116.23 |
| F6 | Ginsenoside Rk2 | 364779-14-6 | 604.90 | 97.02 | 106.88 | 94.83 |
| F7 | Ginsenoside Rh3 | 166040-90-0 | 604.86 | 97.55 | 104.97 | 110.31 |
| F8 | (20S)-Protopanaxdiol | 30636-90-9 | 460.00 | 99.75 | 95.73 | 81.62 |
| F9 | Ginsenoside Rf | 52286-58-5 | 801.01 | 102.49 | 103.60 | 104.31 |
| F10 | Notoginsenoside R2 | 80418-25-3 | 771.00 | 105.59 | 108.58 | 111.17 |
| F11 | (20R)-Protopanaxdiol | 7755-01-3 | 460.74 | 99.62 | 101.25 | 105.48 |
| F12 | 20(R)-Notoginsenoside R2 | 948046-15-9 | 771.00 | 110.54 | 109.60 | 100.25 |
| G1 | Ginsenoside Rg2 | 52286-74-5 | 785.02 | 112.91 | 106.11 | 100.31 |
| G2 | 20(R)-Ginsenoside Rg2 | 80952-72-3 | 785.02 | 104.07 | 104.32 | 122.28 |
| G3 | Ginsenoside Rg6 | 147419-93-0 | 766.50 | 100.32 | 103.59 | 124.68 |
| G4 | Ginsenoside F4 | 181225-33-2 | 767.00 | 99.89 | 89.89 | 99.83 |
| G5 | Ginsenoside Rh1 | 63223-86-9 | 638.88 | 114.23 | 96.54 | 100.34 |
| G6 | Methylophiopogonanone A | 74805-92-8 | 342.34 | 28.87 | 0.52 | 79.90 |
| G7 | Tectorigenin | 548-77-6 | 300.26 | 4.17 | 4.26 | 86.80 |
| G8 | Ipriflavone | 35212-22-7 | 280.32 | 61.30 | 89.52 | 117.21 |
| G9 | Glycyrrhizic acid | 1405-86-3 | 822.93 | 106.02 | 112.28 | 120.99 |
| G10 | Myricitrin | 17912-87-7 | 464.38 | 96.68 | 84.45 | 110.49 |
| G11 | Epibetulinic acid | 38736-77-5 | 456.70 | 105.56 | 111.09 | 120.68 |
| G12 | 8-Methoxypsoralen | 298-81-7 | 216.19 | 106.99 | 110.19 | 119.10 |
| H1 | Diosmetin | 520-34-3 | 300.26 | 0.00 | 56.99 | 39.13 |
| H2 | Bilobetin | 521-32-4 | 552.48 | 23.99 | 0.00 | 42.34 |
| H3 | Palmitic acid | 57-10-3 | 256.42 | 93.07 | 84.80 | 112.73 |
| H4 | (-)-Catechin | 18829-70-4 | 290.27 | 41.36 | 101.25 | 46.83 |
| H5 | Isoxanthohumol | 70872-29-6 | 354.40 | 15.82 | 68.41 | 87.98 |
| H6 | Cardamonin | 19309-14-9 | 270.28 | 71.38 | 31.76 | 88.76 |
| H7 | Schisantherin B | 58546-55-7 | 514.56 | 108.53 | 108.98 | 107.51 |
| H8 | 5,6-Dihydroxy-2-phenylchromen-4-one | 6665-66-3 | 254.24 | 18.14 | 8.07 | 68.42 |
| H9 | Thonningianin A | 271579-11-4 | 874.71 | 11.97 | 3.29 | 17.63 |
| H10 | Epimedin C | 110642-44-9 | 822.80 | 108.62 | 95.77 | 107.48 |
| H11 | Quercetin (positive inhibitor) | 117-39-5 | 302.24 | 0.00 | 0.10 | 39.85 |

| | | | | | | |
|---|---|---|---|---|---|---|
| H12 | Control | - | - | 100.00 | 100.00 | 100.00 |
